# The 2021 WHO catalogue of *Mycobacterium tuberculosis* complex mutations associated with drug resistance: A genotypic analysis

**DOI:** 10.1016/S2666-5247(21)00301-3

**Published:** 2022-04

**Authors:** Timothy M Walker, Paolo Miotto, Claudio U Köser, Philip W Fowler, Jeff Knaggs, Zamin Iqbal, Martin Hunt, Leonid Chindelevitch, Maha Farhat, Daniela Maria Cirillo, Iñaki Comas, James Posey, Shaheed V Omar, Timothy EA Peto, Anita Suresh, Swapna Uplekar, Sacha Laurent, Rebecca E Colman, Carl-Michael Nathanson, Matteo Zignol, Ann Sarah Walker, The CRyPTIC Consortium, Ivan Barilar, Simone Battaglia, Emanuele Borroni, Angela P Brandao, Alice Brankin, Andrea Maurizio Cabibbe, Joshua Carter, Pauline Claxton, David A Clifton, Ted Cohen, Jorge Coronel, Viola Dreyer, Sarah G Earle, Vincent Escuyer, Lucilaine Ferrazoli, George Fu Gao, Jennifer Gardy, Saheer Gharbia, Kelen T Ghisi, Arash Ghodousi, Ana Luíza Gibertoni Cruz, Clara Grazian, Jennifer L Guthrie, Wencong He, Harald Hoffmann, Sarah J Hoosdally, Lisa Jarrett, Lavania Joseph, Ruwen Jou, Priti Kambli, Rukhsar Khot, Anastasia Koch, Thomas Andreas Kohl, Donna Kohlerschmidt, Samaneh Kouchaki, Alexander S Lachapelle, Ajit Lalvani, Louis Grandjean, Simon Grandjean Lapierre, Ian F Laurenson, Brice Letcher, Wan-Hsuan Lin, Chunfa Liu, Dongxin Liu, Kerri M Malone, Ayan Mandal, Daniela Matias, Graeme Meintjes, Flávia F Mendes, Matthias Merker, Marina Mihalic, James Millard, Nerges Mistry, David AJ Moore, Kimberlee A Musser, Dumisani Ngcamu, Nhung N Hoang, Stefan Niemann, Kayzad Soli Nilgiriwala, Camus Nimmo, Nana Okozi, Rosangela S Oliveira, Nicholas I Paton, Juliana MW Pinhata, Sara Plesnik, Zully M Puyen, Marie Sylvianne Rabodoarivelo, Niaina Rakotosamimanana, Paola MV Rancoita, Priti Rathod, Esther Robinson, Gillian Rodger, Camilla Rodrigues, Aysha Roohi, David Santos-Lazaro, Sanchi Shah, E Grace Smith, Walter Solano Lc, Andrea Spitaleri, Philip Supply, Utkarsha Surve, Sabira Tahseen, Nguyen Thuy Thuong Thuong, Guy Thwaites, Katharina Todt, Alberto Trovato, Christian Utpatel, Annelies Van Rie, Srinivasan Vijay, Robin M Warren, Jim Werngren, Robert J Wilkinson, Daniel J Wilson, Pénélope Wintringer, Yu-Xin Xiao, Yang Yang, Zhao Yanlin, Shen-Yuan Yao, Baoli Zhu, The Seq&Treat Consortium, Heidi Albert, Emmanuel Andre, Jason R Andrews, Irena Arandjelovic, Arnold Bainomugisa, Marie Ballif, Anna Barbova, Draurio Barreira, Alain Baulard, Erik C Böttger, Maxine Caws, Angkana Chaiprasert, Darren Chetty, Francesc Coll, Victoria Cook, Teresa Cortes, Christopher Coulter, Helen Cox, Alan Christoffels, Roland Diel, Carla Duncan, Sarah Dunstan, Matthias Egger, Kiatichai Faksri, Margaret M Fitzgibbon, Victoria Furió, Sebastien Gagneux, Patricia J Hall, Tanya A Halse, Zahra Hasan, Kathryn Holt, Michael Inouye, Frances B Jamieson, Babak Javid, James Johnston, Moses Joloba, Danielle Jorgensen, SM Mostofa Kamal, George W Kasule, Peter M Keller, Ellis Kelly, Clare Kong, Julianne V Kus, Pascal Lapierre, Gian Maria Rossolini, Marguerite Massinga Loembé, Vanessa Mathys, Fabrizio Menardo, Alberto Mendoza-Ticona, John Metcalfe, Satoshi Mitarai, Max O’Donnell, Rick Twee-Hee Ong, Sushil Pandey, Amy Piatek, Therdsak Prammananan, Youwen Qin, Leen Rigouts, Jaime Robledo, Mabel Rodrigues, Thomas R Rogers, Emma Roycroft, Vonthanak Saphonn, Marco Schito, Joseph Shea, Vitali Sintchenko, Alena Skrahina, Prapaporn Srilohasin, Adrie JC Steyn, Prapat Suriyaphol, Patrick Tang, Jill Taylor, Dang Thi Minh Ha, Dick van Soolingen, Wayne van Gemert, Phan Vuong Khac Thai, Michael G Whitfield, Mark Wilcox, Timothy William, Teo Yik Ying, Danila Zimenkov, Derrick W Crook, Nazir Ismail, Timothy C Rodwell

**Affiliations:** 1 Nuffield Department of Medicine, University of Oxford, Oxford, UK*; 2 Oxford University Clinical Research Unit, Ho Chi Minh City, Vietnam*; 3 San Raffaele Scientific Institute, Milano, Italy*; 4 Department of Genetics, University of Cambridge, Cambridge, UK; 5 European Bioinformatics Institute, Hinxton, UK*; 6 Imperial College London, UK; 7 Havard Medical School, Boston, USA; 8 Biomedicine Institute of Valencia IBV-CSIC, Spain; 9 CIBER Epidemiology and Public Health, Madrid, Spain; 10 Centres for Disease Control and Prevention, Atlanta, USA*; 11 National Institute for Communicable Diseases, Johannesburg, South Africa*; 12 NIHR Oxford Biomedical Research Centre, Oxford, UK*; 13 The Foundation for Innovative New Diagnostics, Geneva, Switzerland**; 14 Global Tuberculosis Programme, World Health Organization, Geneva, Switzerland*,**; 15 Division of Pulmonary, Critical Care and Sleep Medicine, University of California, San Diego, USA*,**; 1 IRCCS San Raffaele Scientific Institute, Milan, Italy; 2 Oswaldo Cruz Foundation, Rio de Janeiro, Brazil; 3 Institute Adolfo Lutz, São Paulo, Brazil; 4 University of Oxford, Oxford, UK; 5 Stanford University School of Medicine, Stanford, USA; 6 Scottish Mycobacteria Reference Laboratory, Edinburgh, UK; 7 Yale School of Public Health, Yale, USA; 8 Universidad Peruana Cayetano Heredia, Lima, Perú; 9 Wadsworth Center, New York State Department of Health, Albany, USA; 10 Chinese Center for Disease Control and Prevention, Beijing, China; 11 Bill & Melinda Gates Foundation, Seattle, USA; 12 UK Health Security Agency, London, UK; 13 Vita-Salute San Raffaele University, Milan, Italy; 14 University of Sydney, Australia; 15 The University of British Columbia, Vancouver, Canada; 16 Public Health Ontario, Toronto, Canada; 17 SYNLAB Gauting, Munich, Germany; 18 Institute of Microbiology and Laboratory Medicine, IMLred, WHO-SRL Gauting, Germany; 19 EMBL-EBI, Hinxton, UK; 20 National Institute for Communicable Diseases, Johannesburg, South Africa; 21 Public Health England, Birmingham, UK; 22 Taiwan Centers for Disease Control, Taipei, Taiwan; 23 Hinduja Hospital, Mumbai, India; 24 University of Cape Town, Cape Town, South Africa; 25 University of Surrey, Guildford, UK; 26 NIHR Health Protection Research Unit in Respiratory Infections, Imperial College, London, UK; 27 Université de Montréal, Canada; 28 The Foundation for Medical Research, Mumbai, India; 29 Research Center Borstel, Borstel, Germany; 30 Africa Health Research Institute, Durban, South Africa; 31 London School of Hygiene and Tropical Medicine, London, UK; 32 Oxford University Clinical Research Unit, Ho Chi Minh City, Viet Nam; 33 University College London, London, UK; 34 National University of Singapore, Singapore; 35 Instituto Nacional de Salud, Lima, Perú; 36 Institut Pasteur de Madagascar, Antananarivo, Madagascar; 39 Univ. Lille, CNRS, Inserm, CHU Lille, Institut Pasteur de Lille, U1019 - UMR 9017 - CIIL - Center for Infection and Immunity of Lille, F-59000 Lille, France; 40 National TB Reference Laboratory, National TB Control Program, Islamabad, Pakistan; 41 University of Antwerp, Antwerp, Belgium; 42 University of Edinburgh, Edinburgh, UK; 43 SAMRC Centre for Tuberculosis Research, Stellenbosch University, Cape Town, South Africa; 44 Public Health Agency of Sweden, Solna, Sweden; 45 Wellcome Centre for Infectious Diseases Research in Africa, Cape Town, South Africa; 46 Francis Crick Institute, London, UK; 47 Institute of Microbiology, Chinese Academy of Sciences, Beijing, China; 48 German Center for Infection Research (DZIF), Hamburg-Lübeck-Borstel-Riems, Germany; 49 FIND, Cape Town, South Africa; 50 University Hospitals Leuven, Belgium; 51 Stanford University, California, USA; 52 University of Belgrade, Belgrade, Serbia; 53 Queensland Department of Health, Brisbane, Australia; 54 University of Bern, Switzerland; 55 National Institute of phthisiology and pulmonology NAMS Ukraine, Kyiv; 56 Unitaid, Geneva, Switzerland; 57 University of Zürich, Switzerland; 58 Liverpool School of Tropical Medicine, UK; 59 Mahidol University, Thailand; 60 British Columbia Centre for Disease Control, Canada; 61 University of the Western Cape, Bellville, South Africa; 62 University Medical Hospital Schleswig-Holstein, Germany; 63 University of Melbourne, Australia; 64 Irish Mycobacteria Reference Laboratory, Dublin, Ireland; 65 Universitat de València, Spain; 66 Swiss Tropical and Public Health Insitute Basel, Switzerland; 67 The Aga Khan University, Karachi, Pakistan; 68 Monash University, Melbourne, Australia; 69 Baker Institute, Melbourne, Australia; 70 University of California, San Francisco, USA; 71 National TB and Leprosy Programme, Kampala, Uganda; 72 National Institute of Diseases of the Chest and Hospital, Dhaka, Bangladesh; 73 University of Cambridge, Cambridge, UK; 74 Careggi University Hospital, Florence, Italy; 75 Africa CDC, Libreville, Gabon; 76 Belgian Reference Laboratory for *M. tuberculosis*, Sciensano, Belgium; 77 Ministry of Health, Lima, Peru; 78 Research Institute of Tuberculosis, Tokyo, Japan; 79 CAPRISA MRC HIV-TB Pathogenesis and Treatment Research Unit, Durban, South Africa; 80 US Agency for International Development (USAID), Washington, USA; 81 The National Science and Technology Development Agency, Thailand; 82 Institute of Tropical Medicine, Antwerp, Belgium; 83 Corporación Para Investigaciones Biológicas, Universidad Pontificia Bolivariana, Medellín, Colombia; 84 Trinity College Dublin, Ireland; 85 Critical Path Institute, Tucson, Arizona, USA; 86 Republican Scientific and Practical Centre for Pulmonology and TB, Minsk, Belarus; 87 Sidra Medican and Research Center, Doha, Qatar; 88 Pham Ngoc Thach Hospital, Ho Chi Minh City, Vietnam; 89 National Institute for Public Health and the Environment, Bilthoven, The Netherlands; 90 Stop TB Partnership, Geneva Switzerland; 91 Leeds Teaching Hospital NHS Trust, Leeds, UK; 92 Engelhardt Institute of Molecular Biology, Moscow, Russia; 93 University of Health Sciences, Cambodia; 94 Clinical Research Centre, Queen Elizabeth Hospital, Sabah, Malaysia; 95 Centres for Disease Control and Prevention, Atlanta, USA; 96 University of Basel, Basel, Switzerland; 97 Khon Kaen University, Khon Kaen, Thailand; 98 Imperial College, London, UK

## Abstract

**Background:**

Molecular diagnostics are considered the most promising route to achieving rapid, universal drug susceptibility testing for *Mycobacterium tuberculosis*complex (MTBC). We aimed to generate a WHO endorsed catalogue of mutations to serve as a global standard for interpreting molecular information for drug resistance prediction.

**Methods:**

A candidate gene approach was used to identify mutations as associated with resistance, or consistent with susceptibility, for 13 WHO endorsed anti-tuberculosis drugs. 38,215 MTBC isolates with paired whole-genome sequencing and phenotypic drug susceptibility testing data were amassed from 45 countries. For each mutation, a contingency table of binary phenotypes and presence or absence of the mutation computed positive predictive value, and Fisher’s exact tests generated odds ratios and Benjamini-Hochberg corrected p-values. Mutations were graded as Associated with Resistance if present in at least 5 isolates, if the odds ratio was >1 with a statistically significant corrected p-value, and if the lower bound of the 95% confidence interval on the positive predictive value for phenotypic resistance was >25%. A series of expert rules were applied for final confidence grading of each mutation.

**Findings:**

15,667 associations were computed for 13,211 unique mutations linked to one or more drugs. 1,149/15,667 (7·3%) mutations were classified as associated with phenotypic resistance and 107/15,667 (0·7%) were deemed consistent with susceptibility. For rifampicin, isoniazid, ethambutol, fluoroquinolones, and streptomycin, the mutations’ pooled sensitivity was >80%. Specificity was over 95% for all drugs except ethionamide (91·4%), moxifloxacin (91·6%) and ethambutol (93·3%). Only two resistance mutations were classified for bedaquiline, delamanid, clofazimine, and linezolid as prevalence of phenotypic resistance was low for these drugs.

**Interpretation:**

This first WHO endorsed catalogue of molecular targets for MTBC drug susceptibility testing provides a global standard for resistance interpretation. Its existence should encourage the implementation of molecular diagnostics by National Tuberculosis Programmes.

**Funding:**

UNITAID, Wellcome, MRC, BMGF.

## Introduction

Due to disruptions related to the COVID-19 pandemic, it is estimated that in 2020 1.4 million fewer people received treatment for tuberculosis compared to 2019. The estimated additional 500,000 deaths over the previous year’s total of 1.4 million will set the world back to levels of mortality levels not seen since 2010.^1,2^ The diagnosis and appropriate treatment of patients with rifampicin resistance was already challenging pre-pandemic, with fewer than half of an estimated 500,000 patients benefitting.^2^ The availability of several new treatment options and strategies for the first time in decades is an advance, but getting the right drugs to the right patients in time to positively impact outcomes is essential, and requires access to rapid and accurate diagnostics that meet the emerging needs.^3,4^

The World Health Organization (WHO) set an important but challenging target on universal drug susceptibility testing (DST), which includes testing for the new and repurposed drugs in the WHO revised definition of extensively drug-resistant tuberculosis.^5^ Phenotypic DST for *Mycobacterium tuberculosis* complex (MTBC), although still the reference standard for most drugs, can take over a month to complete and requires expensive, complex laboratory capacity. For many countries these challenges remain prohibitive. Genotypic approaches to susceptibility testing can be rapid, accurate, automated and cost-effective.^6^ However, no WHO-endorsed catalogue of mutations for the interpretation of genotypic DST results has hitherto been available.

The combination of rapid and accurate diagnostic tools and supportive WHO policies can have significant global impact.^2^ The WHO previously published a guide to MTBC next-generation sequencing data interpretation, adopting the findings from a previous systematic review of the literature.^7,8^ However, that review did not cover new or repurposed drugs and relied on Sanger sequencing results, which meant that the genomic regions interrogated were inconsistent, increasing the chance of false associations. Gaps in knowledge thus remain. We describe the methods and results of a systematic analysis of a large, globally diverse set of isolates using whole-genome sequencing (WGS) data and accompanying DST, intended to generate more knowledge and create the first WHO endorsed catalogue of mutations for 13 anti-tuberculosis drugs.^9^

## Methods

### Data sources

Existing MTBC WGS data were collected worldwide from academic groups and consortia, reference laboratories, public health organizations and the published literature, along with associated phenotypic DST data (Supplementary Table S1; Appendix). Data were accepted whether locally representative or enriched for resistance. Genomically clustered samples were not excluded, provided these had been assayed independently.

### Phenotypic data

Both categorical (resistant/susceptible) and quantitative (minimum inhibitory concentrations (MICs)) phenotypic data were collected. Different DST methods and resistance-defining critical concentrations were accepted. To ensure comparability and optimise quality, phenotypes were categorised as follows: methods and critical concentrations currently endorsed by WHO (category 1); critical concentrations previously endorsed by WHO for those methods (category 2); methods or critical concentrations not currently endorsed by WHO (category 3). Results in downstream analyses were weighted accordingly (Supplementary Table, S2).

Category 1 methods included Löwenstein-Jensen, Middlebrook 7H10, Middlebrook 7H11, and BACTEC Mycobacterial Growth Indicator Tube (MGIT) by Becton Dickinson, using critical concentrations from current WHO DST guidelines.^10-14^ Phenotypes derived from these media were classified as category 2 if critical concentrations used were outdated, or reported to have relied on WHO guidance without providing the concentration tested.^15-17^ If critical concentrations were unknown, phenotypes were classified as category 3, along with MIC data obtained from Thermo Fisher Scientific broth microdilution plates developed for, and validated by, CRyPTIC, which were converted into categorical results using plate-specific epidemiological cut-offs.^18^ Phenotypic results that did not fit categories 1-3 were excluded.

Where data from more than one phenotypic method were available for an isolate, phenotypes from category 1 were selected over category 2, and these in turn over category 3. Within each category, solid media were ranked above liquid media, which in turn had its own hierarchy, ranking MGIT over microscopic observation drug susceptibility (MODS) over CRyPTIC plates on the basis of historical WHO endorsements.

### Genotypic data

Only WGS data derived from Illumina sequencers were considered. Raw WGS data were processed by Clockwork, a variant calling pipeline developed by CRyPTIC.^19^ As a sanity check and means of measuring quality of the variant calls thereby obtained, 17 well characterised MTBC isolates with high quality polished single-contig hybrid PacBio and Illumina assemblies were used as controls.^19^ These were added to the cohort at the start, and then their variant calls were compared with the truth assemblies following the methodology previously described.^20^ Across these 17 samples, mean precision and recall was 99.8% and 94.7% respectively (after filters, and excluding the untrustworthy masked part of the genome).

### Identification of variants

For each drug, a set of candidate genes and corresponding promoter sequences with a high prior probability of being associated with resistance were identified by an expert panel based on the published literature (Supplementary Table S3). The number of sequence positions not passing a pipeline filter was quantified for each gene for every isolate as a measure of likely local sequencing noise. Assuming a Poisson distribution, where the probability of seeing a given number of calls failing a filter in a given gene was <1% (i.e. a comparatively large number), the phenotype associated with that gene was excluded from further consideration. Isolates with *katG* S315T or *rpoB* S450L variants and susceptible phenotypes to isoniazid (n=128) or rifampicin (n=118), respectively, were also excluded on the assumption that these discrepancies were likely due to sample mislabelling.^21^

An algorithm was then used to categorise the candidate sequences using methods similar to those previously described (Appendix).^22^ The approach reflects the definite defectives method from the field of group testing,^23^ identifying bacterial isolates containing just a single genetic mutation among candidate genes, and associating this with the phenotype.

To maximise the number of isolates in which a mutation can be isolated as the only mutation among candidate genes, a series of pre-processing steps identified mutations consistent with phenotypic susceptibility, and masked these prior to analysis. This was based on the upper bound of the 95% confidence interval on the positive predictive value (PPV) for phenotypic resistance being <10% for any given mutation. Synonymous mutations, loci at otherwise invariant sites for which no base could be called, and variants previously reported as phenotypically neutral were also masked.^24^ Genes were also divided into two tiers for hierarchical analysis, with tier 1 sequences representing those considered by an expert panel to have a higher prior probability of association with resistance, analysed first (Supplementary Table S4).^9^ For isolates with no tier 1 genomic explanation for resistance, tier 2 sequences were analysed.

The algorithmic approach characterised mutations in two passes, as follows: Resistant (‘R’) if a mutation was identified as the only mutation (a ‘solo’) across candidate genes in at least one drug resistant isolate; Susceptible (‘S’) if the variant was only ever seen in susceptible isolates, or only ever in susceptible isolates when solo; or unknown (‘U’) when the variant was never seen solo and not exclusively found in susceptible isolates. Variants characterised as ‘S’ were then masked and the algorithm run a second time (pass 2), identifying additional mutations now exposed as solo and characterising these. Two-by-two tables were generated from the number of susceptible and resistant phenotypes with and without each characterised variant as solo, and odds ratios (OR) and corresponding p-values generated using Fisher’s exact test. Benjamini-Hochberg corrections were used to assess statistical significance using a false discovery rate (FDR) of 5%. PPV and binomial exact 95% confidence intervals were computed from the contingency tables.

The algorithm was run once for category 1 phenotypes, again for category 1 and 2 phenotypes combined, and a third time for all phenotypes together. To avoid perfect prediction for the *katG* S315T or *rpoB* S450L variants (for which any susceptible isolates were excluded assuming sample mislabelling, as described above), phenotypes from excluded isolates were added back in only to compute the OR and PPV for these variants for each iteration.

### Confidence grading

The ORs, PPVs and associated FDR corrected p-values and confidence intervals formed the basis for the confidence grading approach in which variants were assigned to one of five groups: 1) associated with resistance; 2) associated with resistance – interim; 3) uncertain significance; 4) not associated with resistance – interim; 5) not associated with resistance (i.e. ‘consistent with susceptibility’).^25^ Mutations identified as solo with a category 1 or 2 phenotype (i.e. a WHO endorsed method) on at least five occasions; that had a 95% CI lower bound of ≥0.25 for the positive predictive value; and with an OR>1 and a significant FDR-corrected p-value, were classified as ‘associated with resistance’. A mutation was graded as ‘associated with resistance - interim’ if fewer than 5 of the solos were associated with a category 1 or 2 phenotype, or if the mutation was only identified as solo on the second pass of the algorithm.

Mutations were graded as ‘not associated with resistance’ if solos met the pre-processing criteria described above (95% confidence interval upper bound on the positive predictive value for phenotypic resistance <10%). All other mutations were graded as having ‘uncertain significance’.

Unlike many drugs which have hotspots in which resistance mutations cluster in a relevant gene, resistance to pyrazinamide can be conferred by a large number of individually infrequent mutations dispersed across *pncA*.^25^ Confidence grading criteria established for other drugs excluded most of these mutations and were therefore relaxed. Mutations present as solo in *pncA* in at least two resistant isolates and with ≥50% PPV were classified as ‘associated with resistance – interim’, whereas those with a PPV <40% (and upper bound 95% CI <75%) were classified as ‘not associated with resistance – interim’.

Finally, a set of expert rules were applied to mutations of ‘uncertain significance' whereby any nonsynonymous mutation in the rifampicin resistance determining region of *rpoB*, and any premature stop codon or insertion/deletion in *ethA, gid, katG or pncA* was interpreted as ‘associated with resistance – interim’.^8,25^ Mutations not already graded, but for which there was previous guidance from WHO, were graded according to that external evidence, with an ‘interim’ caveat unless these were so-called borderline mutations in the rifampicin resistance determining region, or unless there was more recent evidence in the literature to suggest previous WHO guidance should be revised (see Appendix and detailed methods in WHO document).^9^

### Role of the funding source

The funders of the study had no role in the study design, data collection, data analysis, data interpretation, or writing of the report. The corresponding author had full access to all the data in the study and had final responsibility for the decision to submit for publication.

## Findings

We analysed 41,137 MTBC isolates with phenotypic and WGS data from 41 countries across six continents. Thirty countries contributed data on more than 100 isolates and ten countries contributed more than 1,000 isolates. 38,215 isolates (mean depth of 120x) passed quality control steps and were included in the final analysis.

Not all isolates had phenotypic DST results for all drugs. Data on first-line drugs, isoniazid, rifampicin, ethambutol and pyrazinamide were most common. Phenotypic data on new and repurposed drugs, bedaquiline, delamanid, clofazimine and linezolid, were least common and almost exclusively derived from CRyPTIC plates (Table 1).^18^ The prevalence of resistance to first-line drugs ranged from 14% (pyrazinamide) to 35% (isoniazid). The prevalence of resistance to new (bedaquiline, delamanid) and repurposed (linezolid, clofazimine) drugs was lower than for other drugs (≤1·2%).

**Figure 1:**
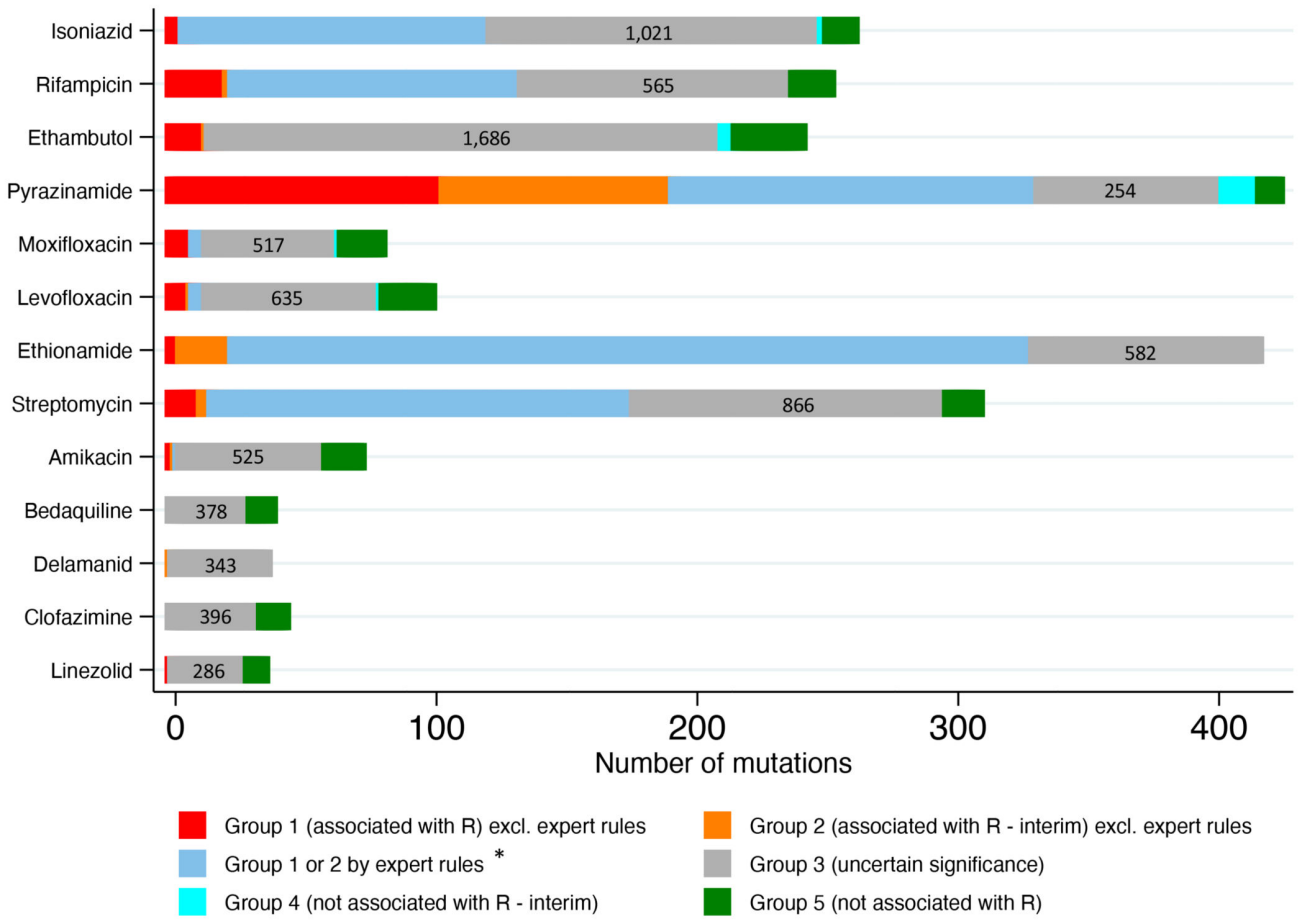
The number of Tier 1 mutations in each group for each drug (no Tier 2 mutations were graded as group 1 or 2 so these are not shown for any groups). To reflect the minimum number of isolates a mutation must have been seen in for it to be graded as group 1 or 2, the mutations counted here are those that were seen in 5 or more isolates. The exceptions are mutations relevant to pyrazinamide (counted if seen in 2 or more isolates) and mutations subject to an expert rule (counted if seen in any number of isolates). The actual number of Tier 1 group 3 mutations (‘Uncertain significance’), regardless of the frequency with which these were seen, is written within each grey bar. Group 4 mutations graded as such by an expert rule are not shown separately here; see Supplementary Table S4 for details on those. *All expert rule mutations were group 2, with the exception of rpoB borderline mutations.

**Figure 2:**
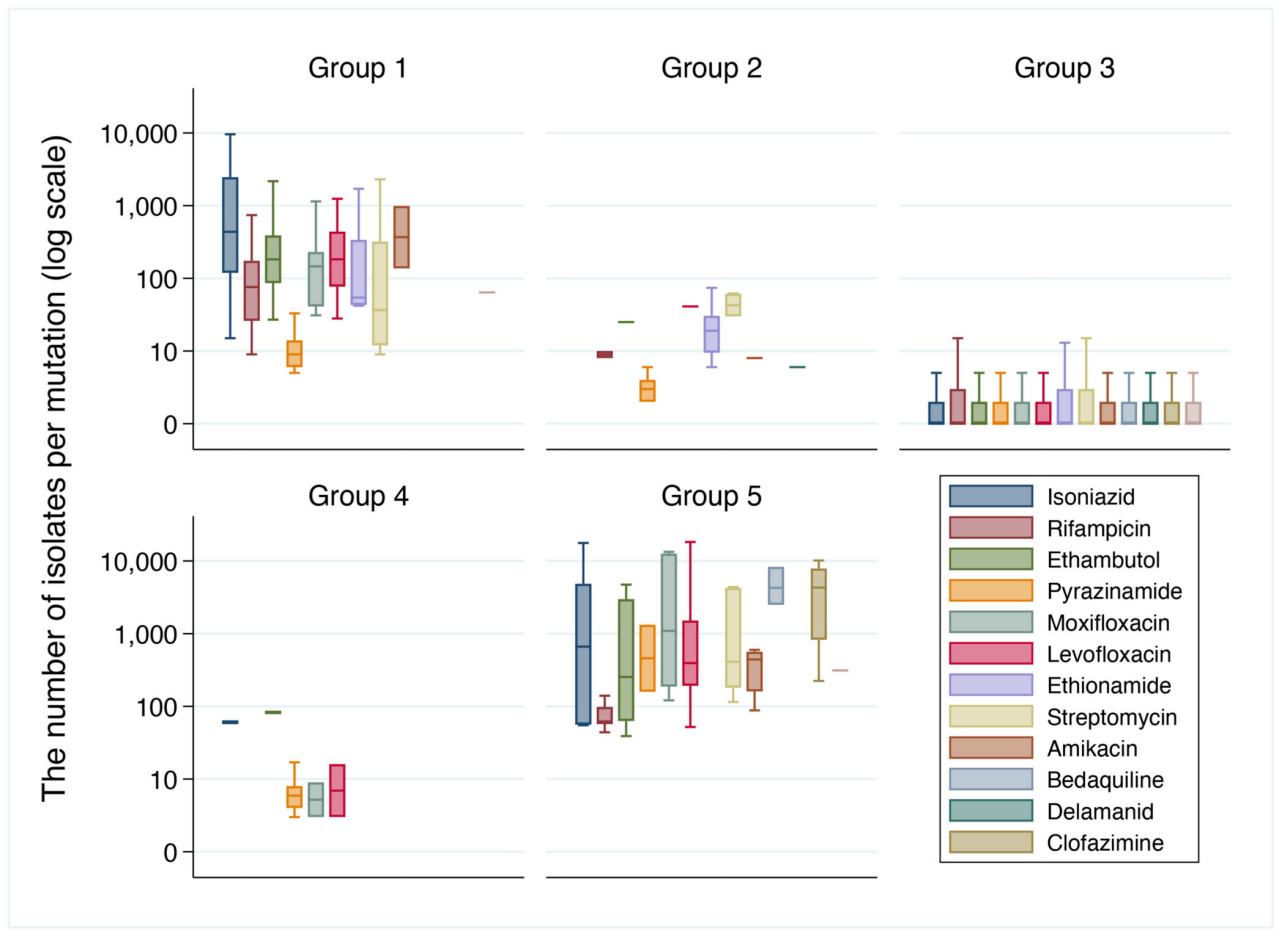
Number of isolates per mutation, by group and drug. Box and whisker plots exclude outside values. Mutations graded by expert rules are not shown as the number of isolates such mutations are seen in is not relevant to their classification. (Group 1 = Associated with Resistance; Group 2 = Associated with Resistance – interim; Group 3 = Uncertain significance; Group 4 = Not associated with Resistance – interim; Group 5 = Not Associated with Resistance

15,667 associations were computed for 13,211 unique mutations relevant to one or more of 13 drugs. 1,149/15,667 (7·3%) mutations were graded as group 1 or 2, and 107/15,667 (0·7%) were graded as group 4 or 5. The majority were graded as of ‘uncertain significance’ (group 3) (Figure 1; Supplementary Table S4). All group 1 and 2 mutations were derived from tier 1 sequences (Supplementary Table S4), and for most drugs from only one or two genes (Supplementary Table S5). Except for *inhA* promoter mutations, all upstream group 1 and 2 mutations were within 12bp of a start codon. Although there were many group 3 mutations, individually these were seen far less frequently than the comparatively small number of group 1 or 2, or group 4 or 5 mutations (Figure 2). For pyrazinamide, only 7% of isolates contained a mutation of uncertain significance, climbing to 42% for bedaquiline (Supplementary Table S6).

**Figure 3:**
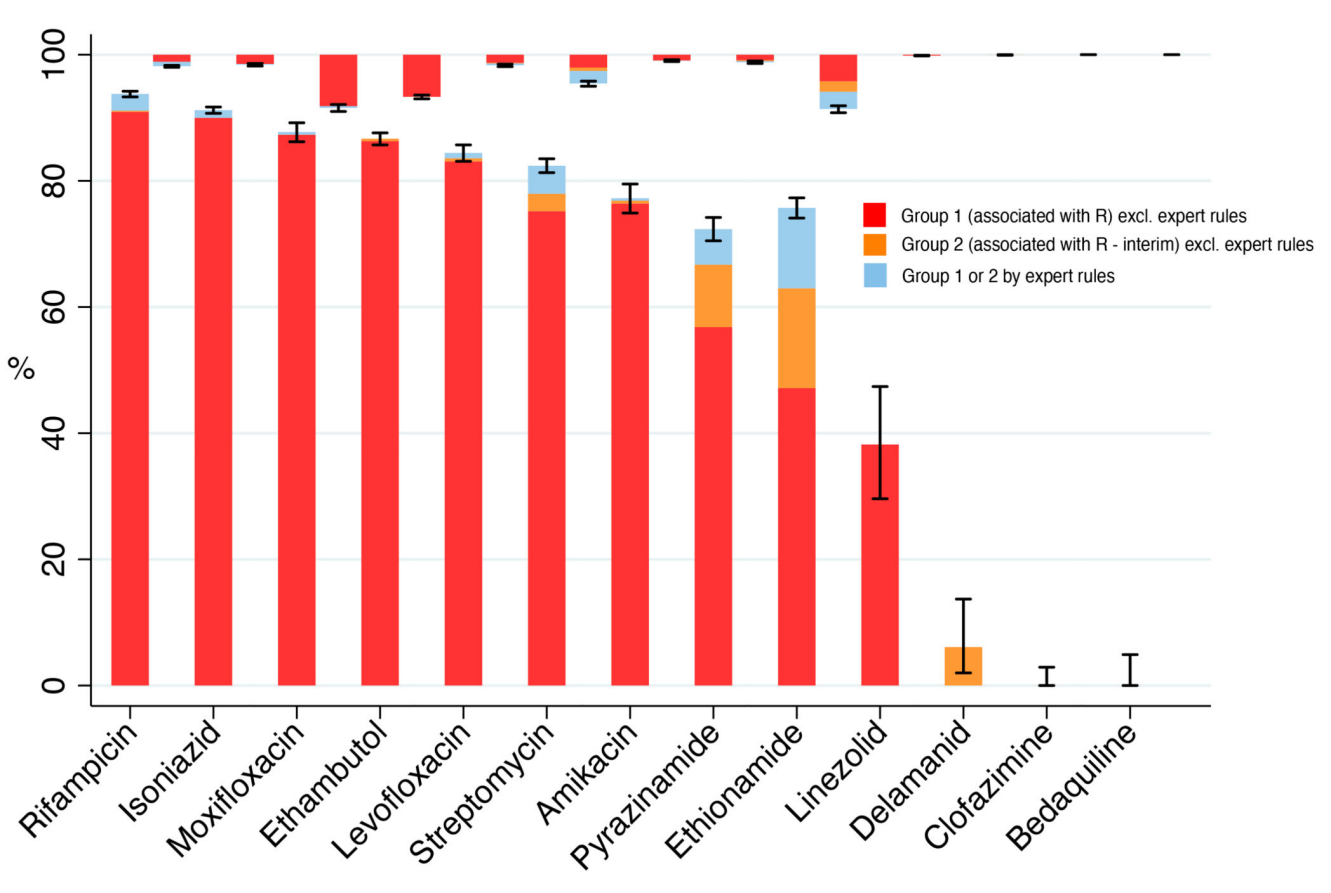
Sensitivity and specificity are shown for all drugs. Sensitivity is represented by bars going upwards from zero, and specificity by bars going downwards from 100. As in figure 1, red and orange represent group 1 (Associated with Resistance) and group 2 (Associated with Resistance – Interim) mutations respectively. Mutations subject to expert rules are separated from their group and shown independently in light blue. The colour scheme shows the incremental sensitivity gained, and corresponding specificity lost, by expanding the catalogue to include first group 1 and then group 2 mutations, in each case without the use of expert rules, and then adding in the expert rules. With the exception of borderline *rpoB* mutations, all mutation subject to an expert rule were graded as group 2. Confidence intervals are for the total effect of all mutations shown.

As no independent dataset was available to test the catalogue, sensitivity and specificity were assessed by predicting phenotypic resistance for the same data from which the catalogue was derived. For rifampicin, isoniazid, ethambutol, fluoroquinolones, and streptomycin, the mutations’ pooled sensitivity was>80%. Specificity was over 95% for all drugs except ethionamide (91·4%), moxifloxacin (91·6%) and ethambutol (93·3%) (Figure 3). In most cases the contribution of expert rules to sensitivity was small. For isoniazid, 10,978/12,199 (90%) resistant isolates contained one of five data-derived resistance mutations, with an additional 148 (1·2%) isolates subject to an expert rule. For rifampicin 9,047/9,868 (91·7%) resistant isolates contained one of the 23 data-derived resistance mutations, with 207 (2·1%) additional isolates subject to expert rules (Figure 3; Supplementary Table S4). For drugs where resistance can be caused by loss of function mutations in non-essential genes, the expert rules played a greater role. For pyrazinamide, 284/2,329 (12·2%) resistant isolates were subject to an expert rule, and for ethionamide 530/2,965 (17·9%) (Supplementary Table S7).

**Figure 4:**
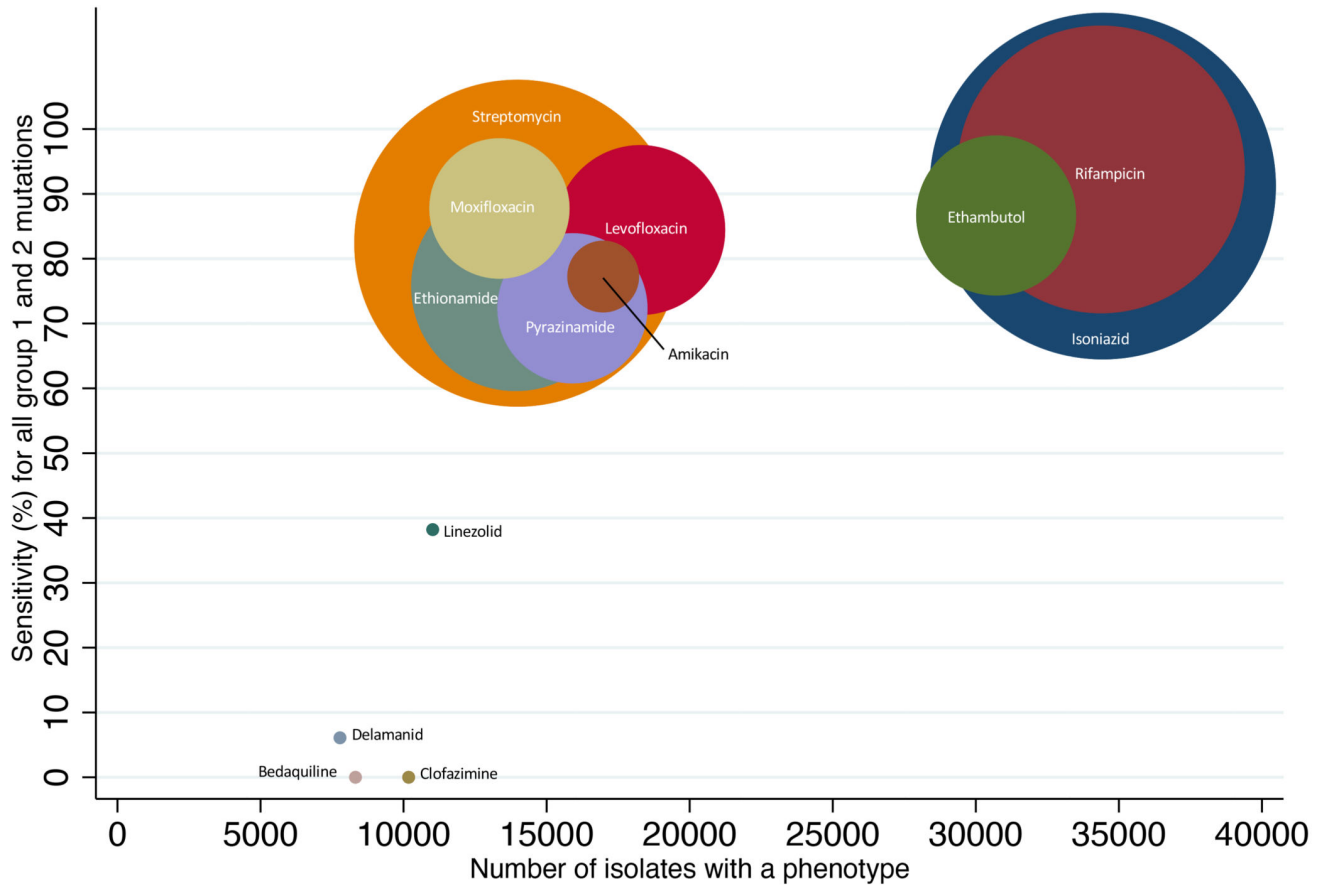
Total number of isolates for each drug plotted against sensitivity. Each drug is represented by a coloured circle that is weighted by the prevalence of phenotypic resistance to that drug in this dataset. The centre of each circle shows the intersection between values on the X and Y axes. Group 1 = Associated with Resistance; Group 2 = Associated with Resistance – interim

Only one mutation was classified as a group 1 resistance associated mutation for linezolid and one group 2 mutation for delamanid. No resistance mutations were identified from the data for bedaquiline or clofazimine. Figure 4 shows how the correspondingly low sensitivity relates to the number of isolates analysed for each drug and to the prevalence of phenotypic resistance among those isolates. Not only were fewer isolates analysed for the novel use drugs, the prevalence of resistance was markedly lower, between 0·9%-1·2%, and ≥2% even among isolates resistant to rifampicin and isoniazid. For legacy-use drugs it was between 7·6%-35·4%.

All genomic and associated phenotypic data are available (see Supplementary Table S1).

## Discussion

This catalogue represents the first WHO endorsed list of genomic mutations associated with drug resistance, or consistent with susceptibility, in MTBC.^9^ It is derived from the largest, globally sourced dataset of MTBC genome sequences and associated phenotypes published to date. This catalogue provides a common starting point and serves as a public resource for a wide array of users from tuberculosis reference laboratories, to molecular diagnostics developers, to surveillance programmes.

The approach adopted to classify mutations combined three previously published schemes that were refined during several rounds of consultation with an international panel of clinicians and researchers.^8,22,25^ The analysis was designed to be deliberately stringent to minimise the chances of having to reverse the grading of any variants in the future. There will therefore be mutations described in the literature, including in known drug resistance genes for new and repurposed drugs, that are not in the catalogue (e.g. *rrl* for linezolid).^3,26^ Indeed, the reported performance of existing catalogues is often higher than the sensitivity and specificity reported here.^21^ The difference is that the WHO catalogue presents robust evidence for each mutation, whereas the sensitivity of other catalogues has benefited from including lower confidence mutations, such as mutations that may only ever have been seen once, or compensatory mutations.^21^ Although the latter can accurately predict resistance (e.g. *ahpC* promotor mutations interrogated by the WHO-endorsed Cepheid Xpert MTB/XDR assay),^9^ this analysis was designed not to associate these with resistance. Nevertheless, as a group lower-confidence mutations are likely to be enriched for resistance, thereby typically improving the sensitivity of a catalogue more than they might negatively impact its specificity. The WHO catalogue may therefore not be the best performing catalogue, but instead provides a common platform from which to build catalogues and diagnostic assay.

This work was not designed as a systematic review of the literature, although decisions by WHO, based on the literature, were incorporated unless more recent literature contradicted these.^9^ Following these precedents, a series of expert rules intended to cover all possible mutations that share functional features, such as premature stop codons, insertions or deletions resulting in the loss of function of non-essential genes such as *katG* or *pncA* were used.^8^ Although the rules are evidence-based, it is possible that exceptions exist. For instance, not all nonsense mutations may result in a loss of function. Expert rules could also have been applied to other non-essential genes relevant to other drugs, but we chose a stringent approach. Rules may need updating as new data accumulate.

While large numbers of isolates can help overcome the variability introduced by random error in measurements, big data remain susceptible to systematic error such as from changes in recommended critical concentrations over time, as happened for the fluroquinolones in 2018.^12^ The phenotypic data here were largely derived from WHO endorsed methods, albeit from a variety of media with varying critical concentrations. The broth microdilution plates by the CRyPTIC consortium are not WHO-endorsed, but contributed phenotypes for over 20% of these isolates, including the vast majority of data for the new and repurposed drugs.^18^ The hierarchical prioritization of phenotypes was an attempt to manage the diversity in phenotypic methods.

Two tiers of candidate genes were analysed, with mainly canonical targets in tier 1 and more recently identified genes in tier 2.^27,28^ No mutations outside of tier 1 genes and their promoters were classified as associated with resistance in this analysis. It could be that tier 2 variants were either too rare, or that the mutations in these genes result in smaller increases in the MIC, producing a more variable binary phenotype that failed to meet the established thresholds.

The specificity of graded mutations was low for some drugs (Figure 3), which is likely due to a combination of four factors.^29^ First, inappropriately high critical concentrations between 2014-18 played a major role for moxifloxacin, despite our hierarchical approach prioritising phenotypic DST results.^10^ Second, some resistance mutations only confer modest MIC increases, which means that their MIC distributions overlap with that of genuinely susceptible isolates. For these mechanisms, phenotypic DST, even using the correct critical concentration, is not a reliable confirmatory method, as recognised by WHO’s endorsement of a composite reference standard for rifampicin.^11^ Third, epistasis could have played a role for amikacin and potentially bedaquiline and clofazimine.^30^ Finally, the expert rules may have overcalled resistance in some cases, as outlined above. It should also be noted that both sensitivity and specificity might be overestimated since these were assessed on the same data from which mutations were graded.

Despite these limitations, progress is to be expected as more resistant isolates are collected. This is especially important for new and repurposed drugs. Diminishing returns are however to be expected from analysing many more isolates resistant to legacy-use drugs, especially where the very major error rate (the gap between observed sensitivity and 100%) starts to overlap with the expected rate of phenotypic or sample labelling error.^21^

There are national tuberculosis programmes that already use WGS in place of phenotypic testing to direct the use of first-line drugs,^21^ but further work is required to expand the catalogue for all drugs, and meet the desired target product profiles for molecular DST.^7,29^ This should focus on collections with more phenotypic resistance to new and repurposed drugs, and could involve *in vivo* and *in vitro* selection experiments.^3^ Future analyses should also focus on the association between mutations, and combinations of mutations, with MICs.^28,29^ Such data would help facilitate the tailoring of individual drug doses based on molecular diagnostics. The WHO plans to regularly update the catalogue and will endeavour to incorporate these advances and address existing gaps to strengthen the public health response. The effort will depend on future contributions from researchers, funding agencies and data custodians to this global effort.

## Supplementary Material

Supplementary tables

Appendix

## Figures and Tables

**Table 1:** Summary of phenotypic DST data reported by drug; number and percentage of the isolates reported with resistant (R) phenotypes.

Drug	Level of Support for CC^a^	Total # Isolates	Total # Resistant	Percentage R (95% CI)
Rifampicin	WHO CURRENT	4,107	1,387	33.8% (32.3-35.2%)
	WHO CURRENT+PAST	27,063	6,736	24.9% (24.4-25.4%)
	ALL	34,375	9,868	28.7% (28.2-29.2%)
Isoniazid	WHO CURRENT	14,252	3,657	25.7% (24.9-26.4%)
	WHO CURRENT+PAST	26,727	8,440	31.6% (31.0-32.1%)
	ALL	34,437	12,199	35.4% (34.9-35.9%)
Ethambutol	WHO CURRENT	11,028	1,307	11.9% (11.3-12.5%)
	WHO CURRENT+PAST	23,706	3,615	15.2% (14.8-15.7%)
	ALL	30,708	4,900	16.0% (15.5-16.4%)
Pyrazinamide	WHO CURRENT	8,416	851	10.1% (9.5-10.8%)
	WHO CURRENT+PAST	15,903	2,329	14.6% (14.1-15.2%)
	ALL^b^	15,902	2,329	14.6% (14.1-15.2%)
Levofloxacin	WHO CURRENT	2,407	194	8.1% (7.0-9.2%)
	WHO CURRENT+PAST	10,305	2,019	19.6% (18.8-20.4%)
	ALL	18,277	3,108	17.0% (16.5-17.6%)
Moxifloxacin	WHO CURRENT	164	12	7.3% (3.8-12.4%)
	WHO CURRENT+PAST	6,904	1,094	15.8% (15.0-16.7%)
	ALL	13,351	1,869	14.0% (13.4-14.6%)
Bedaquiline	WHO CURRENT	0	0	NA
	WHO CURRENT+PAST	88	3	3.4% (0.7-9.6%)
	ALL	8,321	73	0.9% (0.7-1.1%)
Linezolid	WHO CURRENT	72	0	0.0% (0.0-5.0%)
	WHO CURRENT+PAST	1,131	9	0.8% (0.4-1.5%)
	ALL	11,018	123	1.1% (0.9-1.3%)
Clofazimine	WHO CURRENT	0	0	NA
	WHO CURRENT+PAST	3,635	23	0.6% (0.4-0.9%)
	ALL	10,179	125	1.2% (1.0-1.5%)
Delamanid	WHO CURRENT	0	0	NA
	WHO CURRENT+PAST	89	2	2.2% (0.3-7.9%)
	ALL	7,778	82	1.1% (0.8-1.3%)
Amikacin	WHO CURRENT	1,015	60	5.9% (4.5-7.5%)
	WHO CURRENT+PAST	8,040	664	8.3% (7.7-8.9%)
	ALL	16,978	1,288	7.6% (7.2-8.0%)
Streptomycin	WHO CURRENT	1,577	144	9.1% (7.8-10.7%)
	WHO CURRENT+PAST	9,043	2,562	28.3% (27.4-29.3%)
	ALL	13,984	4,635	33.1% (32.4-33.9%)
Ethionamide	WHO CURRENT	45	17	37.8% (23.8-53.5%)
	WHO CURRENT+PAST	2,184	884	40.5% (38.4-42.6%)
	ALL	13,918	2,965	21.3% (20.6-22.0%)

a WHO CURRENT = methods and critical concentrations currently endorsed by WHO (category 1); WHO CURRENT + PAST = category 1 + critical concentrations previously endorsed by WHO for those methods (category 2); ALL = category 1 + category 2 + methods or critical concentrations not currently endorsed by WHO (category 3).

b The 'ALL' dataset has one phenotype fewer than the WHO current and past dataset. This is because a whole strain was removed at the stage when category 3 phenotypes were added. One strain had a category 3 isoniazid phenotye, with a susceptible isoniazid phenotype and a *katG* S315T mutation, but a phenotypes from a higher category for pyrazinamide. The pyrazinamide phenotype was therefore included as part of the WHO current and past dataset. When the isoniazid phenotype was added, and the whole strain was removed, including the pyrazinamide phenotype.

## Research in Context

### Evidence before this study

Much work has been undertaken to describe molecular causes of resistance to anti-tuberculosis drugs over the past three decades. Available tools have changed over the years and whole-genome sequencing (WGS) technology has emerged and taken over from PCR based techniques as the dominant approach to large scale genomic analyses. There have been various independent efforts to consolidate research findings into lists of mutations associated with drug resistance. The TBDreamDB was an early attempt and the systematic review of the literature by the ReSeqTB consortium was a more recent effort. The findings of the latter work were reflected in a WHO guide to the interpretation of *M. tuberculosis* complex (MTBC) WGS data. However, there was no globally endorsed standard of MTBC mutations and interpretation for national tuberculosis programmes or industry to refer to in the design of their services or products.

### Added value of this study

The diverse nature of the data accumulated over the past decades using different technologies and different platforms has made comparability challenging. This study leveraged established knowledge to define a set of candidate genes for each drug and sought to assemble as large a dataset as possible, from as many countries as possible, to perform a *de novo* analysis of all the available WGS data and associated phenotypes. The result is the first WHO endorsed catalogue of mutations associated with drug resistance and consistent with drug susceptibility (published by WHO in 2021) that constitutes an international reference point for national tuberculosis programmes and assay manufacturers. This article reflects the data that generated the catalogue, providing a summary of the findings and an overview of what has been achieved so far, and where future efforts should be invested to improve molecular diagnostics, and thereby also patients care.

### Implication of all the available evidence

The WHO catalogue is a deliberately conservative effort and is not exhaustive. The weight of evidence supporting each mutation in the catalogue should however provide confidence to the tuberculosis community. Great accuracy has been achieved when predicting drug resistance in the past, but not every mutation in previous catalogues has necessarily been as well supported as is the case here. Having a WHO endorsed mutation list should support the field of molecular drug susceptibility testing in MTBC by providing all countries a template for the interpretation of their data, and thereby encouraging adoption of these technologies. WHO plans to update the catalogue on a regular basis, and as more data are generated as a consequence, there are expected to be more data to feed into future analyses, hopefully initiating a virtuous circle.
