# The 2021 WHO catalogue of *Mycobacterium tuberculosis* complex mutations associated with drug resistance: a genotypic analysis

**DOI:** 10.1016/S2666-5247(21)00301-3

**Published:** 2022-04

**Authors:** Timothy M Walker, Paolo Miotto, Claudio U Köser, Philip W Fowler, Jeff Knaggs, Zamin Iqbal, Martin Hunt, Leonid Chindelevitch, Maha R Farhat, Daniela Maria Cirillo, Iñaki Comas, James Posey, Shaheed V Omar, Timothy EA Peto, Anita Suresh, Swapna Uplekar, Sacha Laurent, Rebecca E Colman, Carl-Michael Nathanson, Matteo Zignol, Ann Sarah Walker, Derrick W Crook, Nazir Ismail, Timothy C Rodwell, CRyPTIC Consortium, the Seq&Treat Consortium, A Sarah Walker, Adrie J C Steyn, Ajit Lalvani, Alain Baulard, Alan Christoffels, Alberto Mendoza-Ticona, Alberto Trovato, Alena Skrahina, Alexander S Lachapelle, Alice Brankin, Amy Piatek, Ana Gibertoni Cruz, Anastasia Koch, Andrea Maurizio Cabibbe, Andrea Spitaleri, Angela P Brandao, Angkana Chaiprasert, Anita Suresh, Anna Barbova, Annelies Van Rie, Arash Ghodousi, Arnold Bainomugisa, Ayan Mandal, Aysha Roohi, Babak Javid, Baoli Zhu, Brice Letcher, Camilla Rodrigues, Camus Nimmo, Carl-Michael NATHANSON, Carla Duncan, Christopher Coulter, Christian Utpatel, Chunfa Liu, Clara Grazian, Clare Kong, Claudio U Köser, Daniel J Wilson, Daniela Maria Cirillo, Daniela Matias, Danielle Jorgensen, Danila Zimenkov, Darren Chetty, David AJ Moore, David A Clifton, Derrick W Crook, Dick van Soolingen, Dongxin Liu, Donna Kohlerschmidt, Draurio Barreira, Dumisani Ngcamu, Elias David Santos Lazaro, Ellis Kelly, Emanuele Borroni, Emma Roycroft, Emmanuel Andre, Erik C Böttger, Esther Robinson, Fabrizio Menardo, Flavia F Mendes, Frances B Jamieson, Francesc Coll, George Fu Gao, George W Kasule, Gian Maria Rossolini, Gillian Rodger, E Grace Smith, Graeme Meintjes, Guy Thwaites, Harald Hoffmann, Heidi Albert, Helen Cox, Ian F Laurenson, Iñaki Comas, Irena Arandjelovic, Ivan Barilar, Jaime Robledo, James Millard, James Johnston, Jamie Posey, Jason R Andrews, Jeff Knaggs, Jennifer Gardy, Jennifer Guthrie, Jill Taylor, Jim Werngren, John Metcalfe, Jorge Coronel, Joseph Shea, Joshua Carter, Juliana MW Pinhata, Julianne V Kus, Katharina Todt, Kathryn Holt, Kayzad S Nilgiriwala, Kelen T Ghisi, Kerri M Malone, Kiatichai Faksri, Kimberlee A Musser, Lavania Joseph, Leen Rigouts, Leonid Chindelevitch, Lisa Jarrett, Louis Grandjean, Lucilaine Ferrazoli, Mabel Rodrigues, Maha Farhat, Marco Schito, Margaret M Fitzgibbon, Marguerite Massinga Loembé, Maria Wijkander, Marie Ballif, Marie-Sylvianne Rabodoarivelo, Marina Mihalic, Mark WILCOX, Martin Hunt, Matteo ZIGNOL, Matthias Merker, Matthias Egger, Max O'Donnell, Maxine Caws, Mei-Hua Wu, Michael G Whitfield, Michael Inouye, Mikael Mansjö, Minh Ha Dang Thi, Moses Joloba, SM Mostofa Kamal, Nana Okozi, Nazir ISMAIL, Nerges Mistry, Nhung N Hoang, Niaina Rakotosamimanana, Nicholas I Paton, Paola M V Rancoita, Paolo Miotto, Pascal Lapierre, Patricia J Hall, Patrick Tang, Pauline Claxton, Penelope Wintringer, Peter M Keller, Phan Vuong Khac Thai, Philip W Fowler, Philip Supply, Prapaporn Srilohasin, Prapat Suriyaphol, Priti Rathod, Priti Kambli, Ramona Groenheit, Rebecca E Colman, Rick Twee-Hee Ong, Robin M Warren, Robert J Wilkinson, Roland Diel, Rosangela S Oliveira, Rukhsar Khot, Ruwen Jou, Sabira Tahseen, Sacha Laurent, Saheer Gharbia, Samaneh Kouchaki, Sanchi Shah, Sara Plesnik, Sarah G Earle, Sarah Dunstan, Sarah J Hoosdally, Satoshi Mitarai, Sebastien Gagneux, Shaheed V Omar, Shen-Yuan Yao, Simon Grandjean Lapierre, Simone Battaglia, Stefan Niemann, Sushil Pandey, Swapna Uplekar, Tanya A Halse, Ted Cohen, Teresa Cortes, Therdsak Prammananan, Thomas A Kohl, Nguyen T T Thuong, Tik Ying Teo, Timothy E A Peto, Timothy C Rodwell, Timothy William, Timothy M Walker, Thomas R Rogers, Utkarsha Surve, Vanessa Mathys, Victoria Furió, Victoria Cook, Srinivasan Vijay, Vincent Escuyer, Viola Dreyer, Vitali Sintchenko, Vonthanak Saphonn, Walter Solano, Wan-Hsuan Lin, Wayne van Gemert, Wencong He, Yang Yang, Yanlin Zhao, Youwen Qin, Yu-Xin Xiao, Zahra Hasan, Zamin Iqbal, Zully M Puyen

**Affiliations:** a Nuffield Department of Medicine, University of Oxford, Oxford, UK; b Oxford University Clinical Research Unit, Ho Chi Minh City, Vietnam; c IRCCS San Raffaele Scientific Institute, Milano, Italy; d Department of Genetics, University of Cambridge, Cambridge, UK; e European Bioinformatics Institute, Hinxton, UK; f Imperial College London, London, UK; g Havard Medical School, Boston, MA, USA; h Biomedicine Institute of Valencia IBV-CSIC, Valencia, Spain; i CIBER Epidemiology and Public Health, Madrid, Spain; j Centers for Disease Control and Prevention, Atlanta, GA, USA; k National Institute for Communicable Diseases, Johannesburg, South Africa; l National Institutes for Health Research Oxford Biomedical Research Centre, Oxford, UK; m FIND, Geneva, Switzerland; n Global Tuberculosis Programme, WHO, Geneva, Switzerland; o Division of Pulmonary, Critical Care and Sleep Medicine, University of California, San Diego, CA, USA

## Abstract

**Background:**

Molecular diagnostics are considered the most promising route to achievement of rapid, universal drug susceptibility testing for *Mycobacterium tuberculosis* complex (MTBC). We aimed to generate a WHO-endorsed catalogue of mutations to serve as a global standard for interpreting molecular information for drug resistance prediction.

**Methods:**

In this systematic analysis, we used a candidate gene approach to identify mutations associated with resistance or consistent with susceptibility for 13 WHO-endorsed antituberculosis drugs. We collected existing worldwide MTBC whole-genome sequencing data and phenotypic data from academic groups and consortia, reference laboratories, public health organisations, and published literature. We categorised phenotypes as follows: methods and critical concentrations currently endorsed by WHO (category 1); critical concentrations previously endorsed by WHO for those methods (category 2); methods or critical concentrations not currently endorsed by WHO (category 3). For each mutation, we used a contingency table of binary phenotypes and presence or absence of the mutation to compute positive predictive value, and we used Fisher's exact tests to generate odds ratios and Benjamini-Hochberg corrected p values. Mutations were graded as associated with resistance if present in at least five isolates, if the odds ratio was more than 1 with a statistically significant corrected p value, and if the lower bound of the 95% CI on the positive predictive value for phenotypic resistance was greater than 25%. A series of expert rules were applied for final confidence grading of each mutation.

**Findings:**

We analysed 41 137 MTBC isolates with phenotypic and whole-genome sequencing data from 45 countries. 38 215 MTBC isolates passed quality control steps and were included in the final analysis. 15 667 associations were computed for 13 211 unique mutations linked to one or more drugs. 1149 (7·3%) of 15 667 mutations were classified as associated with phenotypic resistance and 107 (0·7%) were deemed consistent with susceptibility. For rifampicin, isoniazid, ethambutol, fluoroquinolones, and streptomycin, the mutations' pooled sensitivity was more than 80%. Specificity was over 95% for all drugs except ethionamide (91·4%), moxifloxacin (91·6%) and ethambutol (93·3%). Only two resistance mutations were identified for bedaquiline, delamanid, clofazimine, and linezolid as prevalence of phenotypic resistance was low for these drugs.

**Interpretation:**

We present the first WHO-endorsed catalogue of molecular targets for MTBC drug susceptibility testing, which is intended to provide a global standard for resistance interpretation. The existence of this catalogue should encourage the implementation of molecular diagnostics by national tuberculosis programmes.

**Funding:**

Unitaid, Wellcome Trust, UK Medical Research Council, and Bill and Melinda Gates Foundation.

## Introduction

In 2020, an estimated 1·4 million fewer people than in 2019 received treatment for tuberculosis because of disruptions related to the COVID-19 pandemic.^1^ The estimated 1·9 million deaths from tuberculosis in 2020, 500 000 more than in the previous year, will set the world back to levels of mortality not seen since 2010.^1^, ^2^ The diagnosis and appropriate treatment of patients with rifampicin resistance was already a challenge before the pandemic, with less than half of patients benefiting from access.^2^ The availability of several new treatment options and strategies is progress, but getting the right drugs to the right patients in time to influence outcomes positively is essential and requires access to rapid and accurate diagnostics that meet the emerging needs.^3^, ^4^

WHO set an important but challenging target on universal drug susceptibility testing (DST), which includes testing for the new and repurposed drugs in the WHO revised definition of extensively drug-resistant tuberculosis.^5^ Phenotypic DST for *Mycobacterium tuberculosis* complex (MTBC), although still the reference standard for most drugs, can take over a month to complete and requires expensive, complex laboratory capacity. For many countries these challenges remain prohibitive. Genotypic approaches to susceptibility testing can be rapid, accurate, automated, and cost-effective.^6^ However, no WHO-endorsed catalogue of mutations for the interpretation of genotypic DST results is available.

**Research in context**

**Evidence before this study**

We searched PubMed using the search terms “tuberculosis”, “mutation” and “catalogue” (interchanged with “database”) for primary research articles in English from database inception to Feb 19, 2022. We identified publications that used catalogues for drug resistance prediction tools, as well as a catalogue of phylogenetic mutations that was used to inform our work. Among the databases we identified were ones that focused on as few as seven genes, and others that were now outdated. We identified the systematic review of the literature by ReSeqTB, an early precursor of our work, and a second systematic review covering only new and repurposed drugs. There has to date been no WHO endorsed standard of *Mycobacterium tuberculosis* complex (MTBC) mutation and interpretation for national tuberculosis programmes or industry to refer to in the design of their services or products.

**Added value of this study**

The diverse nature of the data accumulated over the past decades using different technologies and different platforms has made comparability challenging. Our study leveraged established knowledge to define a set of candidate genes for each drug and sought to assemble as large a dataset as possible, from as many countries as possible, to do a new analysis of all the available whole-genome sequencing data and associated phenotypes. The result is the first WHO endorsed catalogue of mutations associated with drug resistance and consistent with drug susceptibility (published by WHO in 2021) that constitutes an international reference point for national tuberculosis programmes and assay manufacturers. This analysis reflects the data that generated the catalogue, providing a summary of the findings and an overview of what has been achieved so far, and where future efforts should be invested to improve molecular diagnostics, and thereby also patient care.

**Implications of all the available evidence**

The WHO catalogue is a deliberately conservative effort and is not exhaustive. The weight of evidence supporting each mutation in the catalogue should, however, provide confidence to the tuberculosis community. Great accuracy has been achieved when predicting drug resistance in the past, but not every mutation in previous catalogues has been as well supported as is the case here. Having a WHO endorsed mutation list should support the field of molecular drug susceptibility testing in MTBC by providing all countries a template for the interpretation of their data, and thereby encouraging adoption of these technologies. WHO plans to update the catalogue on a regular basis and, as more data are generated as a consequence, these will feed into future analyses and hopefully initiate a virtuous circle.

The combination of rapid and accurate diagnostic tools and supportive WHO policies can have significant global impact.^2^ WHO previously published a guide to MTBC next-generation sequencing data interpretation, adopting the findings from a previous systematic review of the literature.^7^, ^8^ However, that review did not cover new or repurposed drugs and relied on Sanger sequencing results, which meant that the genomic regions interrogated were inconsistent, increasing the chance of false associations. Gaps in knowledge thus remain. We did a systematic analysis of a large, globally diverse set of isolates using whole-genome sequencing (WGS) data and accompanying DST to generate more knowledge and create the first WHO-endorsed catalogue of mutations for 13 antituberculosis drugs.^9^

## Methods

### Data sources

In this systematic analysis, we collected existing worldwide MTBC WGS data and associated phenotypic DST data from academic groups and consortia, reference laboratories, public health organisations, and published literature (appendix 2 table S1). Data were accepted whether locally representative or enriched for resistance. Samples clustered by genome were not excluded, provided these had been assayed independently.

### Ethics statement

Approval for the CRyPTIC study was obtained from the Taiwan Centers for Disease Control IRB 106209; University of KwaZulu Natal Biomedical Research Ethics Committee (BE022/13); University of Liverpool Central University Research Ethics Committees (2286); Institutional Research Ethics Committee of The Foundation for Medical Research, Mumbai (FMR/IEC/TB/01a/2015 and FMR/IEC/TB/01b/2015); Institutional Review Board of PD Hinduja Hospital and Medical Research Centre, Mumbai (915-15-CR); scientific committee of the Adolfo Lutz Institute (CTC-IAL 47-J/2017); the Ethics Committee (81452517.1.0000.0059) and Ethics Committee review of Universidad Peruana Cayetano Heredia (Lima, Peru); and London School of Hygiene & Tropical Medicine (London, UK).

### Phenotypic data

We collected both categorical (resistant or susceptible) and quantitative (minimum inhibitory concentrations [MICs]) phenotypic data. Different DST methods and resistance-defining critical concentrations were accepted. To ensure comparability and optimise quality, we categorised phenotypes as follows: methods and critical concentrations currently endorsed by WHO (category 1); critical concentrations previously endorsed by WHO for those methods (category 2); methods and critical concentrations not currently or previously endorsed by WHO (category 3). Results in downstream analyses were weighted accordingly (appendix 2 table S2).

Category 1 methods included Löwenstein-Jensen, Middlebrook 7H10, Middlebrook 7H11, and BACTEC Mycobacterial Growth Indicator Tube (MGIT) by Becton Dickinson, using critical concentrations from WHO DST guidelines.^10^, ^11^, ^12^, ^13^, ^14^ Phenotypes derived from these media were classified as category 2 if critical concentrations used were outdated, or reported to rely on WHO guidance without providing the concentration tested.^15^, ^16^, ^17^ If critical concentrations were unknown, phenotypes were classified as category 3, along with MIC data obtained from Thermo Fisher Scientific broth microdilution plates developed for, and validated by, CRyPTIC, which were converted into categorical results using plate-specific epidemiological cutoffs.^18^ Phenotypic results that did not fit categories 1–3 were excluded.

Where data from more than one phenotypic method were available for an isolate, phenotypes from category 1 were selected over category 2, and these in turn over category 3. Within each category, solid media were ranked above liquid media, which in turn had its own hierarchy, ranking MGIT over microscopic observation drug susceptibility over CRyPTIC plates based on historical WHO endorsements.

### Genotypic data

We only considered WGS data derived from Illumina sequencers. Raw WGS data were processed by Clockwork, a variant calling pipeline developed by CRyPTIC.^19^ As a sanity check and means of measuring quality of the variant calls thereby obtained, 17 well characterised MTBC isolates with high quality polished single-contig hybrid PacBio and Illumina assemblies were used as controls.^19^ These were added to the cohort at the start, and then their variant calls were compared with the truth assemblies following the methods previously described.^20^ Across these 17 samples, mean precision was 99·8% and mean recall was 94·7% (after filters, and excluding the untrustworthy masked part of the genome).

### Identification of variants

For each drug, a set of candidate genes and corresponding promoter sequences with a high probability of being associated with resistance were identified by an expert panel based on the published literature (appendix 2 table S3). The number of sequence positions not passing a pipeline filter was quantified for each gene and each isolate as a measure of probable local sequencing noise. Assuming a Poisson distribution, where the probability of a given number of calls failing a filter in a gene was less than 1%, the phenotype associated with that gene was excluded from further consideration. Isolates with *katG* S315T variants and phenotype susceptible to isoniazid (n=128) and isolates with *rpoB* S450L variants and phenotype susceptible to rifampicin (n=118) were also excluded on the assumption that these discrepancies were probably due to sample mislabelling.^21^

An algorithm was then used to categorise the candidate sequences using methods like those previously described (appendix 1 p 8).^22^ Our approach reflects the definite defectives method from the field of group testing;^23^ it identifies bacterial isolates containing just a single genetic mutation in candidate genes and associates this with the phenotype.

To maximise the number of isolates in which a mutation can be isolated as the only mutation in candidate genes, we used a series of pre-processing steps to identify mutations consistent with phenotypic susceptibility and masked these before analysis. These steps were based on the upper bound of the 95% CI on the positive predictive value (PPV) for phenotypic resistance being less than 10% for any given mutation. Synonymous mutations, loci at otherwise invariant sites for which no base could be called, and variants previously reported as phenotypically neutral were also masked.^24^ Genes were also divided into two tiers for hierarchical analysis, in which tier 1 sequences were deemed by an expert panel to have a higher probability of association with resistance, and these were analysed first (appendix 2 table S4).^9^ For isolates with no tier 1 genomic explanation for resistance, tier 2 sequences were analysed.

The algorithmic approach characterised mutations in two passes: resistant if a mutation was identified as the only mutation (a solo mutation) across candidate genes in at least one drug-resistant isolate; susceptible if the variant was only ever seen in susceptible isolates, or only ever in susceptible isolates when solo; or unknown when the variant was never seen solo and not exclusively found in susceptible isolates. Variants characterised as susceptible were then masked and the algorithm run a second time (pass 2) to identify additional mutations now exposed as solo and characterising these. Two-by-two tables were generated from the number of susceptible and resistant phenotypes with and without each characterised variant as solo, and odds ratios (ORs) and corresponding p values generated using Fisher's exact test. Benjamini-Hochberg corrections were used to assess statistical significance using a false discovery rate (FDR) of 5%. PPV and binomial exact 95% CIs were computed from the contingency tables.

The algorithm was run once for category 1 phenotypes, again for category 1 and 2 phenotypes combined, and a third time for all phenotypes together. To avoid perfect prediction for the *katG* S315T or *rpoB* S450L variants (for which any susceptible isolates were excluded assuming sample mislabelling), phenotypes from excluded isolates were added back in only to compute the OR and PPV for these variants for each iteration.

### Confidence grading

The ORs, PPVs, FDR-corrected p values, and CIs formed the basis for the confidence grading approach in which variants were assigned to one of five groups: 1) associated with resistance; 2) associated with resistance—interim; 3) uncertain significance; 4) not associated with resistance—interim; 5) not associated with resistance (ie, consistent with susceptibility).^25^ Mutations were defined as associated with resistance if they were identified as solo with a category 1 or 2 phenotype (ie, a WHO-endorsed method) on at least five occasions, had a 95% CI lower bound of at least 0·25 for the PPV, had an OR of at least 1, and a significant FDR-corrected p value. A mutation was graded as associated with resistance—interim if fewer than 5 of the solos were associated with a category 1 or 2 phenotype, or if the mutation was only identified as solo on the second pass of the algorithm.

Mutations were graded as not associated with resistance if solos met the pre-processing criteria (95% CI upper bound on the PPV for phenotypic resistance <10%). All other mutations were graded as having uncertain significance.

Unlike many drugs that have hotspots in which resistance mutations cluster in a relevant gene, resistance to pyrazinamide can be conferred by many individually infrequent mutations dispersed across *pncA*.^25^ Confidence grading criteria established for other drugs excluded most of these mutations and were therefore relaxed for pyrazinamide. Mutations present as solo in *pncA* in at least two resistant isolates and with at least 50% PPV were classified as associated with resistance—interim, whereas those with a PPV less than 40% (and upper bound 95% CI <75%) were classified as not associated with resistance—interim.

Finally, a set of expert rules were applied to mutations of uncertain significance whereby any non-synonymous mutation in the rifampicin-resistance determining region of *rpoB* and any premature stop codon, insertion, or deletion in *ethA, gid, katG, or pncA* was interpreted as associated with resistance—interim.^8^, ^25^ Mutations not already graded, but for which there was previous guidance from WHO, were graded according to that external evidence, with an interim caveat unless these were so-called borderline mutations in the rifampicin-resistance determining region, or unless there was more recent evidence in the literature to suggest previous WHO guidance should be revised (appendix 1 p 8 and detailed methods in WHO document).^9^

### Role of the funding source

The funders of the study had no role in the study design, data collection, data analysis, data interpretation, or writing of the report.

## Results

We analysed 41 137 MTBC isolates with phenotypic and WGS data from 45 countries across six continents. 30 countries contributed data on more than 100 isolates and ten countries contributed more than 1000 isolates. 38 215 isolates (mean depth of 120×) passed quality control steps and were included in the final analysis.

Not all isolates had phenotypic DST results for all drugs. Data on first-line drugs (isoniazid, rifampicin, ethambutol, and pyrazinamide) were most common. Phenotypic data on new and repurposed drugs (bedaquiline, delamanid, clofazimine, and linezolid) were least common and almost exclusively derived from CRyPTIC plates (table).^18^ The prevalence of resistance to first-line drugs ranged from 14% (pyrazinamide) to 35% (isoniazid). The prevalence of resistance to new (bedaquiline and delamanid) and repurposed (linezolid and clofazimine) drugs was lower than for other drugs (≤1·2%).

**Table:** Number and percentage of isolates reported with resistant phenotypes by drug and level of support for critical concentrations

	**Total isolates (n)**	**Total resistant (n)**	**Percentage with resistant phenotype (95% CI)**
**Rifampicin**			
WHO current*	4107	1387	33·8% (32·3–35·2)
WHO current and past†	27 063	6736	24·9% (24·4–25·4)
All‡	34 375	9868	28·7% (28·2–29·2)
**Isoniazid**			
WHO current	14 252	3657	25·7% (24·9–26·4)
WHO current and past	26 727	8440	31·6% (31·0–32·1)
All	34 437	12 199	35·.4% (34·9–35·9)
**Ethambutol**			
WHO current	11 028	1307	11·9% (11·3–12·5)
WHO current and past	23 706	3615	15·2% (14·8–15·7)
All	30 708	4900	16·0% (15·5–16·4)
**Pyrazinamide**			
WHO current	8416	851	10·1% (9·5–10·8)
WHO current and past	15 903	2329	14·6% (14·1–15·2)
All§	15 902	2329	14·6% (14·1–15·2)
**Levofloxacin**			
WHO current	2407	194	8·1% (7·0–9·2)
WHO current and past	10 305	2019	19·6% (18·8–20·4)
All	18 277	3108	17·0% (16·5–17·6)
**Moxifloxacin**			
WHO current	164	12	7·3% (3·8–12·4)
WHO current and past	6904	1094	15·8% (15·0–16·7)
All	13 351	1869	14·0% (13·4–14·6)
**Bedaquiline**			
WHO current	0	0	NA
WHO current and past	88	3	3·4% (0·7–9·6)
All	8321	73	0·9% (0·7–1·1)
**Linezolid**			
WHO current	72	0	0·0% (0·0–5·0)
WHO current and past	1131	9	0·8% (0·4–1·5)
All	11 018	123	1·1% (0·9–1·3)
**Clofazimine**			
WHO current	0	0	NA
WHO current and past	3635	23	0·6% (0·4–0·9)
All	10 179	125	1·2% (1·0–1·5)
**Delamanid**			
WHO current	0	0	NA
WHO current and past	89	2	2·2% (0·3–7·9)
All	7778	82	1·1% (0·8–1·3)
**Amikacin**			
WHO current	1015	60	5·9% (4·5–7·5)
WHO current and past	8040	664	8·3% (7·7–8·9)
All	16 978	1288	7·6% (7·2–8·0)
**Streptomycin**			
WHO current	1577	144	9·1% (7·8–10·7)
WHO current and past	9043	2562	28·3% (27·4–29·3)
All	13 984	4635	33·1% (32·4–33·9)
**Ethionamide**			
WHO current	45	17	37·8% (23·8–53·5)
WHO current and past	2184	884	40·5% (38·4–42·6)
All	13 918	2965	21·3% (20·6–22·0)

NA=not available.

* Methods and critical concentrations currently endorsed by WHO (category 1).

† Category 1 plus critical concentrations previously endorsed by WHO for those methods (category 2).

‡ Category 1 plus category 2 plus methods or critical concentrations not currently endorsed by WHO (category 3).

§ The all dataset has one phenotype fewer than the WHO current and past dataset because a whole strain was removed at the stage when category 3 phenotypes were added. One strain had a category 3 isoniazid phenotype, with a susceptible isoniazid phenotype and a *katG* S315T mutation, but a phenotype from a higher category for pyrazinamide. The pyrazinamide phenotype was therefore included as part of the WHO current and past dataset. When the isoniazid phenotype was added, the whole strain was removed, including the pyrazinamide phenotype.

**Figure 1:**
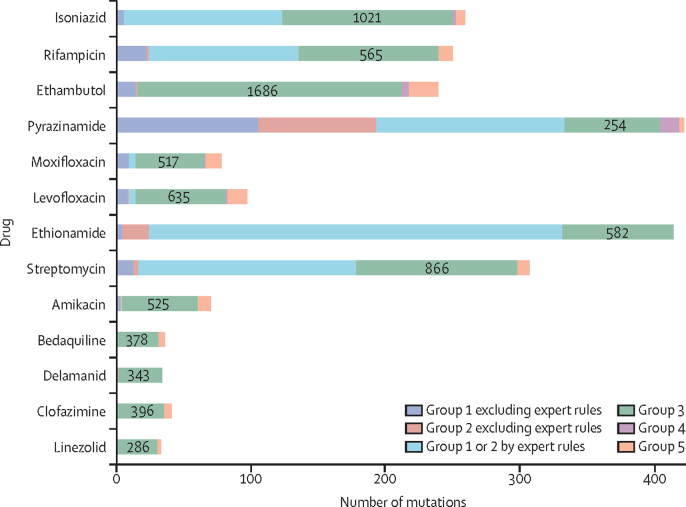
Number of tier 1 mutations by mutation group and drug Genes were divided into two tiers for analysis, in which tier 1 sequences were deemed to have a higher probability of association with resistance. For isolates with no tier 1 genomic explanation for resistance, tier 2 sequences were analysed. No tier 2 mutations were graded as group 1 or 2 so these are not shown for any groups. To reflect the minimum number of isolates a mutation must have been seen in for it to be graded as group 1 or 2, the mutations used here were seen in 5 or more isolates. Exceptions are mutations relevant to pyrazinamide (counted if seen in 2 or more isolates) and mutations subject to an expert rule (counted if seen in any number of isolates). The actual number of tier 1 group 3 mutations, regardless of the frequency with which these were seen, is written within each green bar. Group 4 mutations graded as such by an expert rule are not shown separately here, but can be viewed in appendix 2 (table S4). All expert rule mutations were group 2, with the exception of *rpoB* borderline mutations. Group 1=associated with resistance. Group 2=associated with resistance—interim. Group 3=uncertain significance. Group 4=not associated with resistance—interim. Group 5=not associated with resistance.

**Figure 2:**
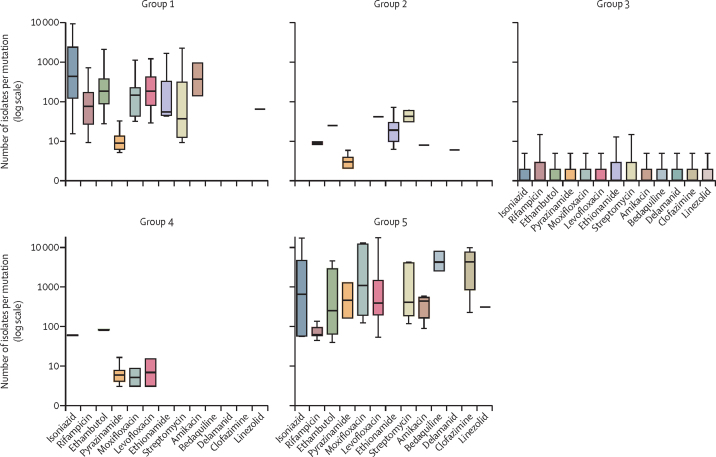
Number of isolates per mutation by mutation group and drug Error bars indicate upper and lower quartiles. Box and whisker plots exclude outside values. Mutations graded by expert rules are not shown as the number of isolates was not relevant to their classification. Group 1=associated with resistance. Group 2=associated with resistance—interim. Group 3=uncertain significance. Group 4=not associated with resistance—interim. Group 5=not associated with resistance.

15 667 associations were computed for 13 211 unique mutations relevant to one or more of 13 drugs. 1149 (7·3%) of 15 667 mutations were graded as group 1 or 2, and 107 (0·7%) were graded as group 4 or 5. Most were graded as of uncertain significance (group 3; figure 1, appendix 2 table S4). All group 1 and 2 mutations were derived from tier 1 sequences (appendix 2 table S4), and for most drugs from only one or two genes (appendix 2 table S5). Except for *inhA* promoter mutations, all upstream group 1 and 2 mutations were within 12 bp of a start codon. Although there were many group 3 mutations, individually these were seen far less frequently than the small number of group 1 or 2, or group 4 or 5 mutations (figure 2). For pyrazinamide, only 7% of isolates contained a mutation of uncertain significance, climbing to 42% for bedaquiline (appendix 2 table S6).

**Figure 3:**
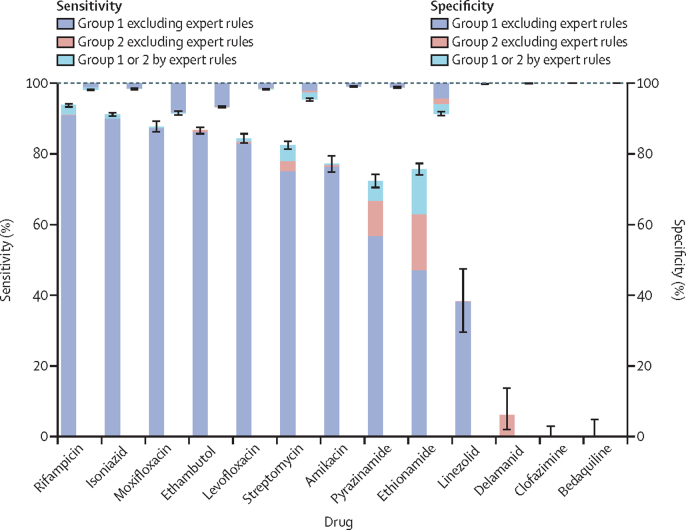
Sensitivity and specificity for all drugs Sensitivity is represented by bars going upwards from zero, and specificity by bars going downwards from 100. The colour progression in each bar shows the incremental sensitivity gained, and corresponding specificity lost, by expanding the catalogue to include first group 1 and then group 2 mutations, in each case without the use of expert rules, and then adding in the expert rules. All mutations subject to an expert rule were graded as group 2 except for borderline *rpoB* mutations. Error bars indicate 95% CIs for the total effect of all mutations shown. Group 1=associated with resistance. Group 2=associated with resistance—interim.

As no independent dataset was available to test the catalogue, sensitivity and specificity were assessed by predicting phenotypic resistance for the same data from which the catalogue was derived. For rifampicin, isoniazid, ethambutol, fluoroquinolones, and streptomycin, the mutations' pooled sensitivity was more than 80%. Specificity was over 95% for all drugs except ethionamide (91·4%), moxifloxacin (91·6%) and ethambutol (93·3%; figure 3). In most cases, the contribution of expert rules to sensitivity was small. For isoniazid, 10 978 (90%) of 12 199 resistant isolates contained one of five data-derived resistance mutations, with an additional 148 (1·2%) isolates subject to an expert rule. For rifampicin, 9047 (91·7%) of 9868 resistant isolates contained one of the 23 data-derived resistance mutations, with 207 (2·1%) additional isolates subject to expert rules (figure 3; appendix 2 table S4). For drugs where resistance can be caused by loss of function mutations in non-essential genes, the expert rules played a greater role. For pyrazinamide, 284 (12·2%) of 2329 resistant isolates were subject to an expert rule, and 530 (17·9%) of 2965 for ethionamide (appendix 2 table S7).

**Figure 4:**
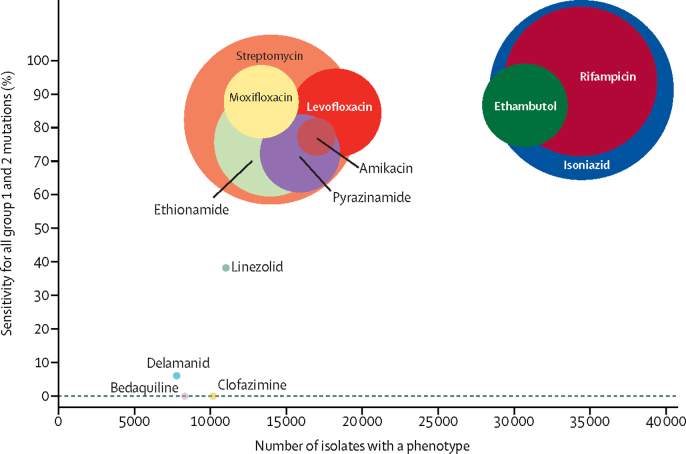
Total number of isolates for each drug by sensitivity Each drug is represented by a coloured circle weighted by the prevalence of phenotypic resistance to that drug. The centre of each circle shows the intersection between values on the x-axis and y-axis. Group 1=associated with resistance. Group 2=associated with resistance—interim.

The only resistance mutations classified for new and repurposed drugs were for linezolid (one mutation classified as group 1) and for delamanid (one mutation classified as group 2). No resistance mutations were identified from the data for bedaquiline or clofazimine. Figure 4 shows how the correspondingly low sensitivity relates to the number of isolates analysed for each drug and to the prevalence of phenotypic resistance in those isolates. Not only were fewer isolates analysed for the novel use drugs, but the prevalence of resistance was also markedly lower, between 0·9–1·2%, and even in isolates resistant to rifampicin and isoniazid, 2% or less. For legacy-use drugs, prevalence of resistance was between 7·6 and 35·4%. All genomic and associated phenotypic data are available in appendix 2 (table S1).

## Discussion

In this genotypic analysis, we present a catalogue that represents the first WHO-endorsed list of genomic mutations associated with drug resistance, or consistent with susceptibility, in MTBC.^9^ The catalogue is derived from the largest, globally sourced dataset of MTBC genome sequences and associated phenotypes published to date. This catalogue provides a common starting point and serves as a public resource for a wide array of users, from tuberculosis reference laboratories, to molecular diagnostics developers, to surveillance programmes.

The approach adopted to classify mutations combined three previously published schemes that were refined during several rounds of consultation with an international panel of clinicians and researchers.^8^, ^22^, ^25^ The analysis was designed to be stringent to minimise the chances of having to reverse the grading of any variants in the future. There will therefore be mutations described in the literature, including in known drug resistance genes for new and repurposed drugs, that are not in the catalogue (eg, *rrl* for linezolid).^3^, ^26^ Indeed, the reported performance of existing catalogues is often higher than the sensitivity and specificity reported here.^21^ However, the WHO catalogue presents robust evidence for each mutation, whereas the sensitivity of other catalogues has benefited from including lower confidence mutations, such as mutations that might only ever have been seen once, or compensatory mutations.^21^ Although compensatory mutations can accurately predict resistance (eg, *ahpC* promotor mutations interrogated by the WHO-endorsed Cepheid Xpert MTB/XDR assay),^9^ this analysis was designed not to associate these with resistance. Nevertheless, collectively, lower confidence mutations are likely to be enriched for resistance, thereby improving the sensitivity of a catalogue more than negatively affecting its specificity. The WHO catalogue might therefore not be the best performing catalogue, but provides a common platform from which to build catalogues and diagnostic assay.

This work was not designed as a systematic review of the literature, although decisions by WHO were incorporated unless more recent literature contradicted these.^9^ Following these precedents, we used a series of expert rules intended to cover all possible mutations that share functional features such as premature stop codons, insertions, or deletions resulting in the loss of function of non-essential genes such as *katG* or *pncA*.^8^ Although the rules are evidence-based, exceptions might exist. For example, not all nonsense mutations will necessarily result in a loss of function. Expert rules could also have been applied to other non-essential genes relevant to other drugs, but we chose a stringent approach. Rules might need to be updated as new data accumulate.

Although many isolates can help overcome the variability introduced by random error in measurements, big data remains susceptible to systematic error, such as from changes in recommended critical concentrations over time, as happened for the fluoroquinolones in 2018.^12^ The phenotypic data were largely derived from WHO-endorsed methods, albeit from various media with varying critical concentrations. The broth microdilution plates used by the CRyPTIC consortium are not WHO-endorsed, but contributed phenotypes for over 20% of these isolates, including almost all data for the new and repurposed drugs.^18^ The hierarchical prioritisation of phenotypes was an attempt to manage the diversity in phenotypic methods.

Two tiers of candidate genes were analysed, with mainly canonical targets in tier 1 and more recently identified genes in tier 2.^27^, ^28^ No mutations outside of tier 1 genes and their promoters were classified as associated with resistance in this analysis. It could be that tier 2 variants were either too rare or that the mutations in these genes result in smaller increases in the MIC, producing a more variable binary phenotype that failed to meet the established thresholds.

The specificity of graded mutations was low for some drugs (figure 3), which is probably due to a combination of four factors.^29^ First, inappropriately high critical concentrations between 2014 and 2018 will have had a major influence on results for moxifloxacin, despite our hierarchical approach prioritising phenotypic DST results.^10^ Second, some resistance mutations only confer modest MIC increases, which means that their MIC distributions overlap with that of genuinely susceptible isolates. Even using the correct critical concentration, phenotypic DST is not a reliable confirmatory method for these mechanisms, as recognised by WHO endorsement of a composite reference standard for rifampicin.^11^ Third, epistasis could have played a role for amikacin and potentially bedaquiline and clofazimine.^30^ Finally, the expert rules might have overcalled resistance in some cases. It should also be noted that both sensitivity and specificity might be overestimated since these were assessed on the same data from which mutations were graded.

Despite these limitations, progress is to be expected as more resistant isolates are collected. This is especially important for new and repurposed drugs. Diminishing returns are to be expected from analysing many more isolates resistant to legacy-use drugs, especially where the very major error rate (the gap between observed sensitivity and 100%) starts to overlap with the expected rate of phenotypic or sample labelling error.^21^

Some national tuberculosis programmes already use WGS in place of phenotypic testing to direct the use of first-line drugs,^21^ but further work is required to expand the catalogue for all drugs and meet the desired target product profiles for molecular DST.^7^, ^29^ This additional work should focus on collections with more phenotypic resistance to new and repurposed drugs, and could involve in vivo and in vitro selection experiments.^3^ Future analyses should also focus on the association between mutations, and combinations of mutations, with MICs.^28^, ^29^ Such data would help facilitate the tailoring of individual drug doses based on molecular diagnostics. WHO plans to update the catalogue regularly and will endeavour to incorporate these advances and address existing gaps to strengthen the public health response. The effort will depend on future contributions from researchers, funding agencies, and data custodians to this global effort.

Other members of the CRyPTIC Consortium and of the Seq&Treat Consortium are listed in appendix 1

## Data sharing

All data used in this study are available in the tables presented as supplementary materials. European Nucleotide Archive accession numbers are provided for the raw sequencing data for all isolates in appendix 2, table S1.

## Declaration of interests

CUK is a consultant for Becton Dickinson, FIND, and the TB Alliance. CUK is collaborating with Janssen, PZA Innovation, and Thermo Fisher Scientific; worked as a consultant for QuantuMDx, the Stop TB Partnership, the WHO Global TB Programme, and the WHO Regional Office for Europe; and gave a paid educational talk for Oxford Immunotec. Hain Lifescience covered CUK's travel and accommodation to present at a meeting. CUK is an unpaid adviser to BioVersys and GenoScreen. ERR is employed by the UK Health Security Agency (UKHSA) and holds an honorary contract with Imperial College London. IFL is director of the Scottish Mycobacteria Reference Laboratory. SN receives funding from German Center for Infection Research, Excellenz Cluster Precision Medicine in Chronic Inflammation, and Leibniz Science Campus Evolutionary Medicine of the LUNG (EvoLUNG)tion EXC 2167. PS is a consultant at Genoscreen. TR is funded by the National Institutes for Health and US Department of Defence and receives salary support from the non-profit organisation FIND. TR is a cofounder, board member, and shareholder of Verus Diagnostics, a company that was founded with the intent of developing diagnostic assays. Verus Diagnostics was not involved in any way with data collection, analysis, or publication of the results, and TR has not received any financial support from Verus Diagnostics. University of California, San Diego (UCSD) Conflict of Interest office has reviewed and approved TR's role in Verus Diagnostics. TR is a coinventor of a provisional patent for a TB diagnostic assay (provisional patent 63/048.989). TR is a coinventor on a patent associated with the processing of tuberculosis sequencing data (European Patent Application 14840432.0 & USSN 14/912,918). TR has agreed to “donate all present and future interest in and rights to royalties from this patent” to UCSD to ensure that he does not receive any financial benefits from this patent. SS works and holds stock options at HaystackAnalytics (Product: Using whole genome sequencing for drug susceptibility testing for *Mycobacterium tuberculosis*). GFG is listed as an inventor on patent applications for RBD-dimer-based coronavirus vaccines. The patents for these RBD dimers as protein subunit vaccines for SARS-CoV-2 have been licensed to Anhui Zhifei Longcom Biopharmaceutical. IC is a consultant for FIND. DAC reports funding from GlaxoSmithKline and consultancy fees from Biobeats, Oxford University Innovation, and Sensyne Health. CC reports funding from FIND to his institution (Pathology Queensland, Queensland Department of Health) for his laboratory to perform molecular analytic studies (limits of detection) for new molecular platforms manufactured by Cepheid and Bioneer. The other authors declare no competing interests.
