# Supplementary material for: The 2021 WHO catalogue of Mycobacterium tuberculosis complex mutations associated with drug resistance: a genotypic analysis
Source: Lancet Microbe. 2022 Apr;3(4):e265–73. doi: 10.1016/S2666-5247(21)00301-3 (PMC7612554; doi:10.1016/S2666-5247(21)00301-3)
Supplement: Supplementary appendix 1 [file mmc1.pdf]

THE LANCET
Microbe

Supplementary appendix 1

This appendix formed part of the original submission and has been peer reviewed. We post it as supplied by the authors.

Supplement to: Walker TM, Miotto P, Köser CU, et al. The 2021 WHO catalogue of *Mycobacterium tuberculosis* complex mutations associated with drug resistance: a genotypic analysis. *Lancet Microbe* 2022; published online March 8. https://doi.org/10.1016/S2666-5247(21)00301-3

Appendix

Contributing through the CRyPTIC consortium (in alphabetical order)

Ivan Barilar MSc,[29] Simone Battaglia PhD[1], Emanuele Borroni ScD[1], Angela P Brandao MSc[2,3], Alice Brankin[4], Andrea Maurizio Cabibbe PhD[1], Joshua Carter[5], Darren Chetty[30], Pauline Claxton MSc[6], Prof David A Clifton DPhil[4], Prof Ted Cohen DPH[7], Jorge Coronel MSc[8], Viola Dreyer Dr.rer.nat.[29], Sarah G Earle Dphil[4], Vincent Escuyer PhD[9], Lucilaine Ferrazoli PhD[3], Prof George Fu Gao DPhil[10], Jennifer Gardy PhD[11], Saheer Gharbia PhD[12], Kelen T Ghisi BSc[3], Arash Ghodousi PhD[1,13], Ana Luíza Gibertoni Cruz[4], Clara Grazian PhD[14], Ramona Groenheit PhD[44], Jennifer L Guthrie PhD[15,16], Wencong He[10], Harald Hoffmann MD[17,18], Sarah J Hoosdally PhD[4], Lisa Jarrett MSc[21], Lavania Joseph[20], Prof Ruwen Jou PhD[22], Priti Kambli MSc[23], Rukhsar Khot[23], Anastasia Koch PhD[24], Thomas Andreas Kohl Dr.rer.nat[29], Donna Kohlerschmidt BS[9], Samaneh Kouchaki PhD[4,25], Alexander S Lachapelle MD[4], Prof Ajit Lalvani[26], Louis Grandjean MBBS[33], Simon Grandjean Lapierre MD[27], Ian F Laurenson FRCPath[6], Brice Letcher MSc[19], Wan-Hsuan Lin MS[22], Chunfa Liu[10], Dongxin Liu[10], Kerri M Malone PhD[19], Ayan Mandal MSc[28], Mikael Masjö MSc[44], Daniela Matias,[21] Prof Graeme Meintjes[24], Flávia F Mendes BSc[3], Matthias Merker Dr.rer.nat[29], Marina Mihalic,[18] James Millard BMBS[30,102], Nerges Mistry PhD[28], Prof David AJ Moore MD[31,8], Kimberlee A Musser PhD[9], Dumisani Ngcamu[20], Nhung N Hoang MSc[32], Prof Stefan Niemann Dr.rer.nat.[29,48], Kayzad Soli Nilgiriwala PhD[28], Camus Nimmo PhD[30,33], Max O'Donnell MD[99], Nana Okozi[20], Rosangela S Oliveira PhD[3], Prof Nicholas I Paton MD[34], Juliana MW Pinhata PhD[3], Sara Plesnik[18], Zully M Puyen PhD[35], Marie Sylvianne Rabodoarivelo[36], Niaina Rakotosamimanana PhD[36], Paola MV Rancoita[13], Priti Rathod MSc[21], Esther R Robinson DPhil,[21] Gillian Rodger PhD[4], Camilla Rodrigues MD[23], Aysha Roohi PhD[4], David Santos-Lazaro MSc[35], Sanchi Shah MSc[28], E Grace Smith FRCPath[21,12], Walter Solano Lc[8], Andrea Spitaleri PhD[1,13], Philip Supply PhD[39], Adrie JC Steyn PhD[30], Utkarsha Surve[23], Sabira Tahseen[40], Nguyen Thuy Thuong Thuong PhD[4,32], Prof Guy Thwaites FMedSci[32,4], Katharina Todt BSc, Alberto Trovato[1], Christian Utpatel[29], Prof Annelies Van Rie[41], Srinivasan Vijay PhD[42], Prof Robin M Warren PhD[43], Jim Werngren PhD[44], Maria Wijkander MSc[44], Prof Robert J Wilkinson FMedSci[45,46,98], Daniel J Wilson DPhil[4], Pénélope Wintringer MSc[19], Yu-Xin Xiao MS[22], Yang Yang[4,101], Zhao Yanlin[10], Shen-Yuan Yao[20], Baoli Zhu[47]

(CRyPTIC authors named on the title page are identified there by a double dagger against their affiliation)

Contributing through the Seq&Treat consortium (in alphabetical order)

Heidi Albert[49], Emmanuel Andre[50], Jason R Andrews[51], Irena Arandjelovic PhD[52], Arnold Bainomugisa PhD[53], Marie Ballif[54], Anna Barbova PhD[55], Draurio Barreira[56], Alain Baulard PhD[39], Prof Erik C Böttger MD[57], Maxine Caws PhD[58], Prof Angkana Chaiprasert Dr.rer.nat.[59], Francesc Coll[31], Victoria Cook MD[60], Teresa Cortes PhD[31], Christopher Coulter[53], Helen Cox PhD[24], Prof Alan Christoffels PhD[61], Prof Roland Diel PhD[62], Carla Duncan BSc[16], Sarah Dunstan PhD[63], Prof Matthias Egger MD[54], Kiatichai Faksri PhD[97], Margaret M Fitzgibbon PhD[64], Victoria Furió PhD[65], Prof Sebastien Gagneux PhD[66,96], Patricia J Hall,[95] Tanya A Halse[9], Prof Zahra Hasan PhD[67], Kathryn Holt PhD[68], Michael Inouye PhD[69], Frances B Jamieson MD[16,103], Babak Javid PhD[70], James Johnston MPH[60], Moses Joloba[71], Danielle Jorgensen BSc[60], Prof SM Mostofa Kamal[72], George W Kasule[71], Peter M Keller[54], Ellis Kelly BSc[73], Clare Kong BSc[60], Julianne V Kus PhD[16,103], Pascal Lapierre PhD[9], Gian Maria Rossolini[74], Marguerite Massinga

Loembé PhD[75], Vanessa Mathys PhD[76], Fabrizio Menardo PhD[66,96], Alberto Mendoza-Ticona[77], Prof John Metcalfe MD[70], Prof Satoshi Mitarai DPhil[78], Rick Twee-Hee Ong [34], Sushil Pandey PhD[53], Amy Piatek[80], Therdsak Prammananan Dr.der.hum.biol.[81], Youwen Qin[63,69], Leen Rigouts PhD[82], Jaime Robledo PhD[83], Mabel Rodrigues PhD[60], Prof Thomas R Rogers DMed[64,84], Emma Roycroft[64], Prof Vonthanak Saphonn[93], Marco Schito[85], Joseph Shea MS[9], Prof Vitali Sintchenko[14], Alena Skrahina MD[86], Prapaporn Srilohasin PhD[59], Prapat Suriyaphol PhD[59], Patrick Tang PhD[87], Jill Taylor PhD[9], Dang Thi Minh Ha[88], Prof Dick van Soolingen PhD[89], Wayne van Gemert[90], Phan Vuong Khac Thai[88], Michael G Whitfield PhD[41,98], Prof Mark Wilcox MD[91,100], Timothy William FRCP[94], Prof Yik Ying Teo[34], Danila Zimenkov PhD[92]
(Seq&Treat authors named on the title page are identified there by a silcrow against their affiliation)

1 IRCCS San Raffaele Scientific Institute, Milan, Italy
2 Oswaldo Cruz Foundation, Rio de Janeiro, Brazil
3 Adolfo Lutz Institute, São Paulo, Brazil
4 University of Oxford, Oxford, UK
5 Stanford University School of Medicine, Stanford, USA
6 Scottish Mycobacteria Reference Laboratory, Edinburgh, UK
7 Yale School of Public Health, Yale, USA
8 Universidad Peruana Cayetano Heredia, Lima, Perú
9 Wadsworth Center, New York State Department of Health, Albany, USA
10 Chinese Center for Disease Control and Prevention, Beijing, China
11 Bill & Melinda Gates Foundation, Seattle, USA
12 UK Health Security Agency, London, UK
13 Vita-Salute San Raffaele University, Milan, Italy
14 University of Sydney, Australia
15 The University of British Columbia, Vancouver, Canada
16 Public Health Ontario, Toronto, Canada
17 SYNLAB Gauting, Munich, Germany
18 Institute of Microbiology and Laboratory Medicine, IMLred, WHO-SRL Gauting, Germany
19 EMBL-EBI, Hinxton, UK
20 National Institute for Communicable Diseases, Johannesburg, South Africa
21 UK Health Security Agency, Birmingham, UK
22 Taiwan Centers for Disease Control, Taipei, Taiwan
23 Hinduja Hospital, Mumbai, India
24 University of Cape Town, Cape Town, South Africa
25 University of Surrey, Guildford, UK
26 NIHR Health Protection Research Unit in Respiratory Infections, Imperial College, London, UK
27 Université de Montréal, Canada
28 The Foundation for Medical Research, Mumbai, India
29 Research Center Borstel, Borstel, Germany
30 Africa Health Research Institute, Durban, South Africa
31 London School of Hygiene and Tropical Medicine, London, UK
32 Oxford University Clinical Research Unit, Ho Chi Minh City, Viet Nam
33 University College London, London, UK

34 Saw Swee Hock School of Public Health, National University of Singapore, Singapore
35 Instituto Nacional de Salud, Lima, Perú
36 Institut Pasteur de Madagascar, Antananarivo, Madagascar
39 Univ. Lille, CNRS, Inserm, CHU Lille, Institut Pasteur de Lille, U1019 - UMR 9017 - CIIL - Center for Infection and Immunity of Lille, F-59000 Lille, France
40 National TB Reference Laboratory, National TB Control Program, Islamabad, Pakistan
41 University of Antwerp, Antwerp, Belgium
42 University of Edinburgh, Edinburgh, UK
43 SAMRC Centre for Tuberculosis Research, Stellenbosch University, Cape Town, South Africa
44 Public Health Agency of Sweden, Solna, Sweden
45 Wellcome Centre for Infectious Diseases Research in Africa, Cape Town, South Africa
46 Francis Crick Institute, London, UK
47 Institute of Microbiology, Chinese Academy of Sciences, Beijing, China
48 German Center for Infection Research (DZIF), Hamburg-Lübeck-Borstel-Riems, Germany
49 FIND, Cape Town, South Africa
50 University Hospitals Leuven, Belgium
51 Stanford University, California, USA
52 University of Belgrade, Belgrade, Serbia
53 Queensland Mycobacterium Reference Laboratory, Brisbane, Queensland, Australia
54 University of Bern, Switzerland
55 National Institute of phthisiology and pulmonology NAMS Ukraine, Kyiv
56 Unitaid, Geneva, Switzerland
57 University of Zürich, Switzerland
58 Liverpool School of Tropical Medicine, UK
59 Mahidol University, Thailand
60 British Columbia Centre for Disease Control, Canada
61 South African MRC Bioinformatics Unit, University of the Western Cape, Bellville, South Africa
62 University Medical Hospital Schleswig-Holstein, Germany
63 University of Melbourne, Australia
64 Irish Mycobacteria Reference Laboratory, Dublin, Ireland
65 Universitat de València, Spain
66 Swiss Tropical and Public Health Insitute Basel, Switzerland
67 The Aga Khan University, Karachi, Pakistan
68 Monash University, Melbourne, Australia
69 Baker Institute, Melbourne, Australia
70 University of California, San Francisco, USA
71 National TB and Leprosy Programme, Kampala, Uganda
72 National Institute of Diseases of the Chest and Hospital, Dhaka, Bangladesh
73 University of Cambridge, Cambridge, UK
74 Careggi University Hospital, Florence, Italy
75 Africa CDC, Libreville, Gabon
76 Belgian Reference Laboratory for *M. tuberculosis*, Sciensano, Belgium
77 Ministry of Health, Lima, Peru
78 Research Institute of Tuberculosis, Tokyo, Japan
79 CAPRISA MRC HIV-TB Pathogenesis and Treatment Research Unit, Durban, South Africa

80 US Agency for International Development (USAID), Washington, USA
81 The National Science and Technology Development Agency, Thailand
82 Institute of Tropical Medicine, Antwerp, Belgium
83 Corporación Para Investigaciones Biológicas, Universidad Pontificia Bolivariana, Medellín, Colombia
84 Clinical Microbiology, Trinity College Dublin, Dublin, Ireland
85 Critical Path Institute, Tucson, Arizona, USA
86 Republican Scientific and Practical Centre for Pulmonology and TB, Minsk, Belarus
87 Sidra Medicine, Doha, Qatar
88 Pham Ngoc Thach Hospital, Ho Chi Minh City, Vietnam
89 National Institute for Public Health and the Environment, Bilthoven, The Netherlands
90 Stop TB Partnership, Geneva Switzerland
91 Leeds Teaching Hospital NHS Trust, Leeds, UK
92 Engelhardt Institute of Molecular Biology, Moscow, Russia
93 University of Health Sciences, Cambodia
94 Clinical Research Centre, Queen Elizabeth Hospital, Sabah, Malaysia
95 US Centers for Disease Control and Prevention, Atlanta, USA
96 University of Basel, Basel, Switzerland
97 Khon Kaen University, Khon Kaen, Thailand
98 Imperial College, London, UK
99 Colombia University Irving Medical Center, New York, NY, USA
100 University of Leeds, Leeds, UK
101 School of Public Health, Shanghai Jiao Tong University School of Medicine, China
102 University of Liverpool, Liverpool, UK
103 Department of Laboratory Medicine and Pathobiology, University of Toronto, Toronto, Canada

Acknowledgments – funders

This work was supported by Wellcome Trust/Newton Fund-MRC Collaborative Award (200205/Z/15/Z); and Bill & Melinda Gates Foundation Trust (OPP1133541); and supported by a grant from Unitaid. This work was funded by National Institutes of Health/ National Institute of Allergy and Infectious Disease (NIH/NIAID R01 AI55765 to MRF). J.A. has received funding from NIH (R01 AI130058). The NIH/NIAID had no role in study design, data collection, and interpretation, or the decision to submit the work for publication. Oxford CRyPTIC consortium members are funded/supported by the National Institute for Health Research (NIHR) Oxford Biomedical Research Centre (BRC), the views expressed are those of the authors and not necessarily those of the NHS, the NIHR or the Department of Health, and the National Institute for Health Research (NIHR) Health Protection Research Unit in Healthcare Associated Infections and Antimicrobial Resistance, a partnership between Public Health England and the University of Oxford, the views expressed are those of the authors and not necessarily those of the NIHR, Public Health England or the Department of Health and Social Care. J.M. is supported by the Wellcome Trust (203919/Z/16/Z). Z.Y. is supported by the National Science and Technology Major Project, China Grant No. 2018ZX10103001. K.M.M. is supported by EMBL's EIPOD3 programme funded by the European Union’s Horizon 2020 research and innovation programme under Marie Skłodowska Curie Actions. T.C.R. is funded in part by funding from Unitaid Grant No. 2019-32-FIND MDR. R.S.O. is supported by FAPESP Grant No. 17/16082-7. L.F. received financial support from FAPESP Grant No. 2012/51756-5.

B.Z. is supported by the National Natural Science Foundation of China (81991534) and the Beijing Municipal Science & Technology Commission (Z201100005520041). N.T.T.T. is supported by the Wellcome Trust International Intermediate Fellowship (206724/Z/17/Z). G.T. is funded by the Wellcome Trust. R.W. is supported by the South African Medical Research Council. J.C. is supported by the Rhodes Trust and Stanford Medical Scientist Training Program (T32 GM007365). T.C. has received grant funding and salary support from US NIH, CDC, USAID and Bill and Melinda Gates Foundation. I.C. laboratory is supported by ERC CoG 101001038 and Ministerio de Ciencia project PID2019-104477RB-I00 L.G. was supported by the Wellcome Trust (201470/Z/16/Z), the National Institute of Allergy and Infectious Diseases of the National Institutes of Health under award number 1R01AI146338, the GOSH Charity (VC0921) and the GOSH/ICH Biomedical Research Centre (www.nihr.ac.uk). A.L. is supported by the National Institute for Health Research (NIHR) Health Protection Research Unit in Respiratory Infections at Imperial College London. J.R. is funded by MinCiencias, Colombia (No. 221389666216 CT-783-2018). S.G.L. is supported by the Fonds de Recherche en Santé du Québec. C.N. is funded by Wellcome Trust Grant No. 203583/Z/16/Z. A.V.R. is supported by Research Foundation Flanders (FWO) under Grant No. G0F8316N (FWO Odysseus). G.M. was supported by the Wellcome Trust (098316, 214321/Z/18/Z, and 203135/Z/16/Z), and the South African Research Chairs Initiative of the Department of Science and Technology and National Research Foundation (NRF) of South Africa (Grant No. 64787). The funders had no role in the study design, data collection, data analysis, data interpretation, or writing of this report. The opinions, findings and conclusions expressed in this manuscript reflect those of the authors alone. A.B. is funded by the NDM Prize Studentship from the Oxford Medical Research Council Doctoral Training Partnership and the Nuffield Department of Clinical Medicine. L.C. acknowledges funding from the MRC Centre for Global Infectious Disease Analysis (reference MR/R015600/1), jointly funded by the UK Medical Research Council (MRC) and the UK Foreign, Commonwealth & Development Office (FCDO), under the MRC/FCDO Concordat agreement and is also part of the EDCTP2 programme supported by the European Union. D.J.W. is supported by a Sir Henry Dale Fellowship jointly funded by the Wellcome Trust and the Royal Society (Grant No. 101237/Z/13/B) and by the Robertson Foundation. A.S.W. is an NIHR Senior Investigator. T.M.W. is a Wellcome Trust Clinical Career Development Fellow (214560/Z/18/Z). C.U.K. is a research associate at Wolfson College and visiting scientist at the Department of Genetics, University of Cambridge. C.U.K. received an observership by the European Society of Clinical Microbiology and Infectious Diseases. I.C. is supported by ERC CoG 101001038 and Ministerio de Ciencia project PID2019-104477RB-I00. A.S.L. is supported by the Rhodes Trust. M.G.W is supported by the National Institute for Health Research (NIHR) Health Protection Research Unit in Healthcare Associated Infections and Antimicrobial Resistance at Imperial College London in partnership with UK Health Security Agency (UKHSA) in collaboration with, Imperial Healthcare Partners, University of Cambridge and University of Warwick. R.J.W. receives funding from the Francis Crick Institute which is supported by Wellcome Trust, (FC0010218), UKRI (FC0010218), and CRUK (FC0010218). The computational aspects of this research were supported by the Wellcome Trust Core Award Grant Number 203141/Z/16/Z and the NIHR Oxford BRC. Parts of the work were funded by the German Center of Infection Research (DZIF). The Scottish Mycobacteria Reference Laboratory is funded through National Services Scotland. The Wadsworth Center contributions were supported in part by Cooperative Agreement No. U60OE000103 funded by the Centers for Disease Control and Prevention through the Association of Public Health Laboratories and NIH/NIAID grant AI-117312.

Additional support for sequencing and analysis was contributed by the Wadsworth Center Applied Genomic Technologies Core Facility and the Wadsworth Center Bioinformatics Core. SYNLAB Holding Germany GmbH for its direct and indirect support of research activities in the Institute of Microbiology and Laboratory Medicine Gauting. N.R. thanks the Programme National de Lutte contre la Tuberculose de Madagascar. A grant from the National University of Singapore Yong Loo Lin School of Medicine Aspiration Fund (NUHSRO/2014/069/AF-New Idea/04, to Drs. Ong and Teo). The US National Institutes of Health, National Institute of Allergy and Infectious Diseases, the Eunice Kennedy Shriver National Institute of Child Health and Human Development, the National Cancer Institute, the National Institute of Mental Health, the National Institute on Drug Abuse, the National Heart, Lung, and Blood Institute, the National Institute on Alcohol Abuse and Alcoholism, the National Institute of Diabetes and Digestive and Kidney Diseases, the Fogarty International Center, and the National Library of Medicine: Asia-Pacific, U01AI069907; CCASAnet, U01AI069923; Central Africa, U01AI096299; East Africa, U01AI069911; NA-ACCORD, U01AI069918; Southern Africa, U01AI069924; West Africa, U01AI069919. The Swiss National Science Foundation (grant number 320030_153442, 189498).

1. Countries from which isolates were derived:

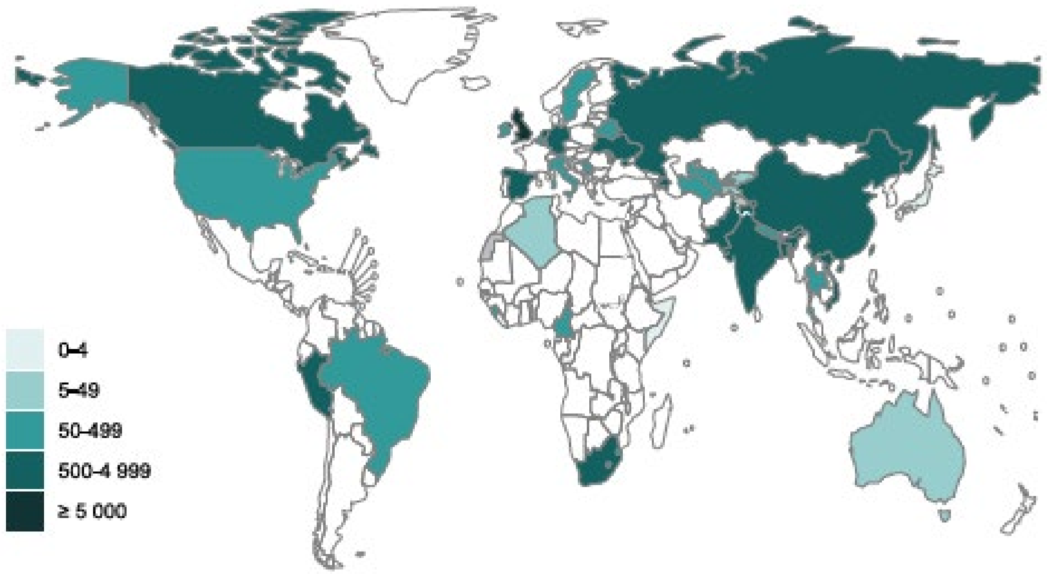

Shadings represent the number of isolates from each country. Figure originally published in https://www.who.int/publications/i/item/9789240028173 **(reference 9).**

2. Figure illustrating the steps to identify neutral mutations, before the algorithm was run:

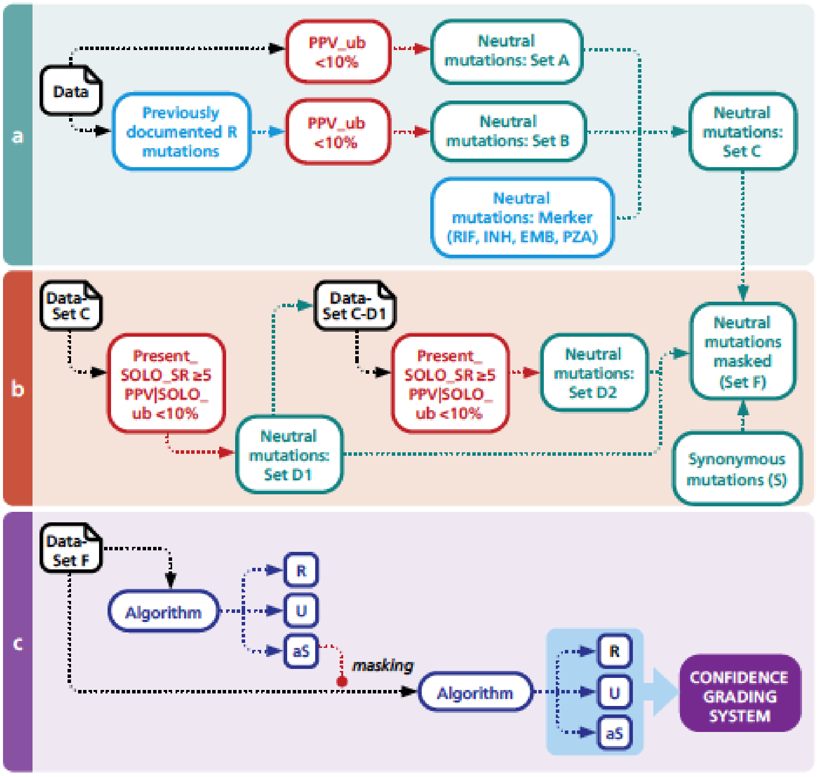

Figure originally published in https://www.who.int/publications/i/item/9789240028173 **(reference 9).**

3. Flow diagram illustrating how the algorithm classified variants:

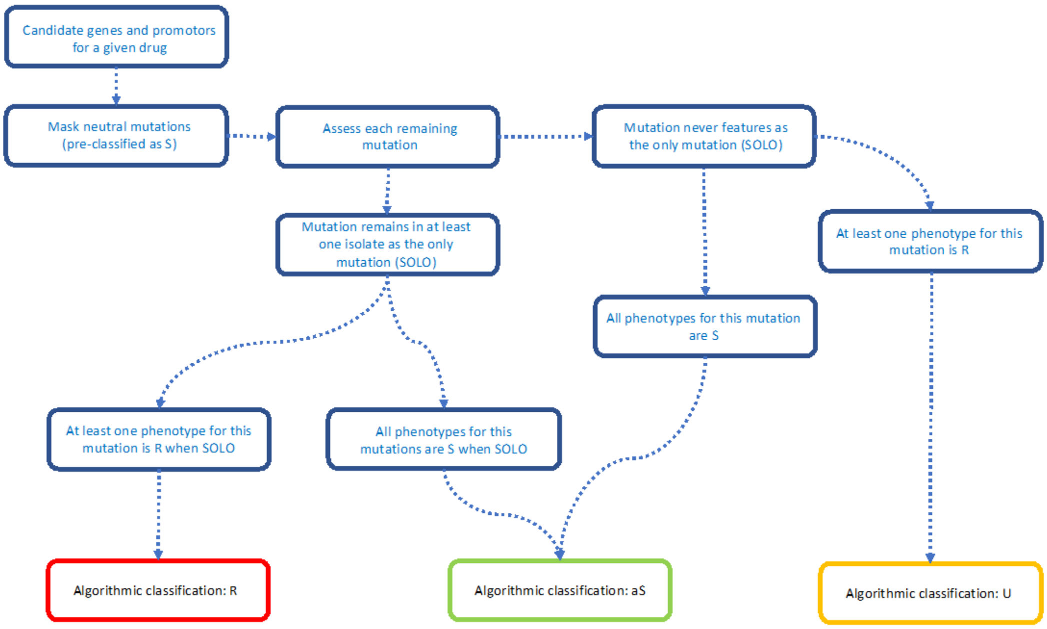

4. Figure illustrating the confidence grading of mutations identified by the algorithm:

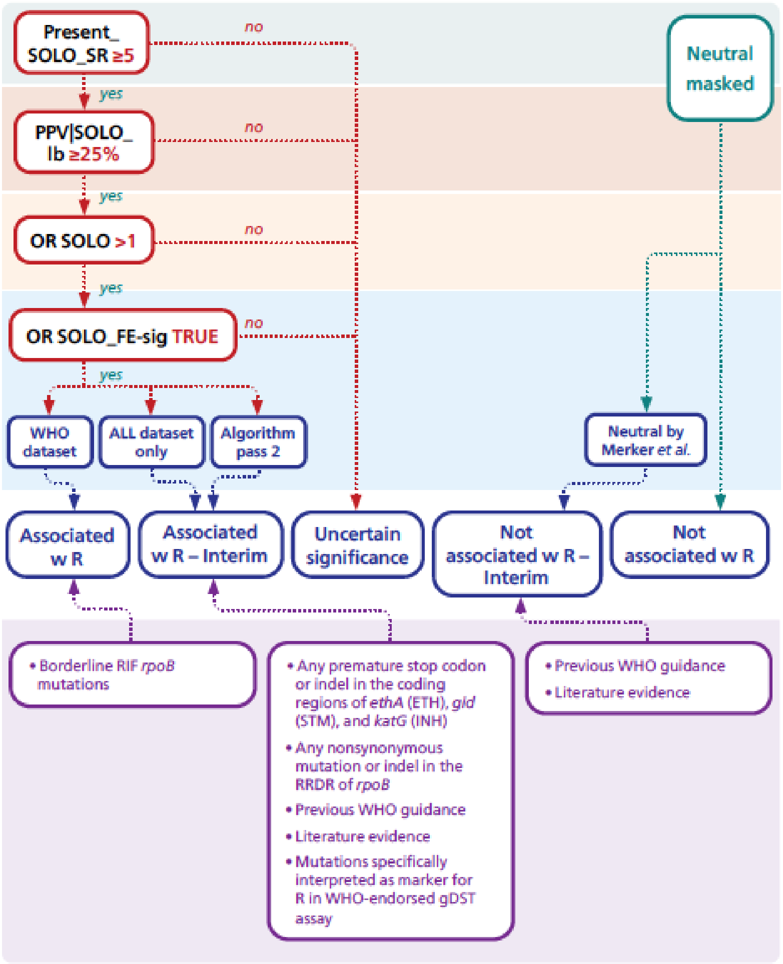

Figure originally published in https://www.who.int/publications/i/item/9789240028173 **(reference 9).**
