# Supplementary material for: The 2021 WHO catalogue of Mycobacterium tuberculosis complex mutations associated with drug resistance: A genotypic analysis
Source: Lancet Microbe. Author manuscript; Available in PMC 2022 Apr 1. (PMC7612554; doi:10.1016/S2666-5247(21)00301-3)
Supplement: Supplementary file 1 [file EMS140756-supplement-Appendix.docx]

Appendix

1. Countries from which isolates were derived:

Shadings represent the number of isolates from each country. Figure originally published in [**https://www.who.int/publications/i/item/9789240028173**](https://www.who.int/publications/i/item/9789240028173) **(reference 9).**

2. Figure illustrating the steps to identify neutral mutations, before the algorithm was run:

Figure originally published in [**https://www.who.int/publications/i/item/9789240028173**](https://www.who.int/publications/i/item/9789240028173) **(reference 9).**

3. Flow diagram illustrating how the algorithm classified variants:

4. Figure illustrating the confidence grading of mutations identified by the algorithm:

Figure originally published in [**https://www.who.int/publications/i/item/9789240028173**](https://www.who.int/publications/i/item/9789240028173) **(reference 9).**
